# Arctic Clouds and Precipitation in the Community Earth System Model Version 2

**DOI:** 10.1029/2020JD032521

**Published:** 2020-11-20

**Authors:** Elin A. McIlhattan, Jennifer E. Kay, Tristan S. L'Ecuyer

**Affiliations:** ^1^ Department of Atmospheric and Oceanic Sciences University of Wisconsin‐Madison Madison WI USA; ^2^ Department of Atmospheric and Oceanic Sciences University of Colorado Boulder CO USA; ^3^ Cooperative Institute for Research in Environmental Sciences University of Colorado Boulder CO USA

**Keywords:** Arctic, clouds, precipitation, CESM2, CAM6

## Abstract

The Arctic climate is changing rapidly, warming at about twice the rate of the planet. Global climate models (GCMs) are invaluable tools both for understanding the drivers of these changes and predicting future Arctic climate evolution. While GCMs are continually improving, there remain difficulties in representing cloud processes which occur on scales smaller than GCM resolution. Since clouds influence the Arctic energy and water cycles, their accurate representation in models is critical for robust future projections. In this work, we examine the representation of Arctic clouds and precipitation in the Community Earth System Model (CESM) with the Community Atmosphere Model (CAM), comparing the newly released version (CESM2 with CAM6) with its predecessor (CESM1 with CAM5). To isolate changes in the Arctic mean state, we compare preindustrial control runs. Arctic cloud ice has decreased slightly, while cloud water has increased dramatically in CESM2. Annual mean liquid‐containing cloud (LCC) frequency has increased from 19% in CESM1 to 51% in CESM2. Since LCCs strongly modulate downwelling radiation at the surface, their increase has led to an increase in mean downwelling longwave (+22 W m^−2^) and corresponding decrease in downwelling shortwave (−23 W m^−2^) radiation. The mean Arctic surface temperature increased from 257 K in CESM1 to 260 K in CESM2, with the largest seasonal difference in winter (+6 K). Annual average snowfall has decreased slightly (−1 mm month^−1^), while rainfall has increased (+5 mm month^−1^).

## Key Points

- The mean Arctic state in CESM2 is cloudier, warmer, and rainier than in CESM1
- Arctic cloud liquid water path is five times greater in CESM2 relative to CESM1
- Arctic rainfall increases in CESM2 relative to CESM1, occurring year‐round across the region in the new version

## 1 Introduction

The Arctic climate is undergoing rapid change (Serreze & Barry, 2011). Observations show that sea ice thickness and extent are decreasing (Onarheim et al., 2018), the Greenland Ice Sheet (GrIS) is losing mass (Mouginot et al., 2019), and permafrost is melting (Schuur et al., 2015), all beyond what is expected from natural variability. Global climate models (GCMs) are essential tools for understanding the mechanisms driving these deviations and for simulating possible future scenarios which aid communities in planning and preparing for longer term climate changes.

As far back as 1896, Arrhenius was able to use relatively simple models and calculations to predict that increased carbon dioxide in the Earth's atmosphere would lead to global surface temperature increases, with enhanced warming of the high latitudes (Arrhenius, 1896). In the intervening years between Arrhenius' prediction and our ability to observe global warming, simple physical models (Budyko, 1969) and early GCMs (Manabe & Stouffer, 1980) continued to highlight the Arctic as a focal point for increased temperatures, due in large part to the ice albedo feedback— ice reflects more incoming solar radiation than ocean or land surfaces, so as the ice melts, more radiation is absorbed leading to more warming and more melt.

With advances in computational capabilities, modern GCMs have swiftly increased in complexity. This has led to models with more detailed representations of real world processes and forecasts and a better ability to reproduce the present‐day climate in agreement with observations (Knutti et al., 2013). However, GCMs still struggle to represent some processes that occur on scales smaller than their spatial resolution. Differences between how individual GCMs parameterize subscale process contribute to the persistent, large intermodel spread for societally important predictions such as the magnitude of future warming (Knutti & Sedláček, 2012) and the onset of ice‐free Arctic summers (Stroeve & Notz, 2015). Clouds in particular evolve and change rapidly on small spatial scales and so must be parameterized in GCMs. Clouds influence Earth's water budget through precipitation and Earth's energy budget by modulating the solar energy that reaches the surface and trapping terrestrial radiation that would otherwise escape to space. Clouds and their feedbacks were specifically identified in the Intergovernmental Panel on Climate Change (IPCC) Fifth Assessment Report (AR5) as one of the major remaining challenges in accurately modeling future climate scenarios (IPCC, 2013).

The Community Earth System Model version 1 (CESM1) was one of the GCMs included in IPCC AR5 as part of the Phase 5 Coupled Model Intercomparison Project (CMIP5). CESM1 contains fully coupled atmosphere, ocean, land, and sea ice components that together simulate Earth's past, present, and future climate. Among the models included in CMIP5, CESM1 showed the closest agreement to global observations of temperature and precipitation when examining interannual variations and the seasonal cycle (Knutti et al., 2013). All components of CESM1 were recently updated by their respective modeling teams and a new wave model was added, resulting in the release of version 2 (CESM2). The atmospheric component was updated from the Community Atmospheric Model version 5 (CAM5) to version 6 (CAM6) (Gettelman et al., 2019).

While CAM5 overall represents Earth's atmosphere relatively well (Knutti et al., 2013), its representation of the Arctic atmosphere has some known issues. Relative to observations, CAM5 does not contain enough Arctic clouds (English et al., 2014; Kay et al., 2012). Supercooled liquid‐containing clouds (LCCs) are underrepresented in CAM5's Arctic (Cesana et al., 2015; Kay, Bourdages et al., 2016; McIlhattan et al., 2017; Tan & Storelvmo, 2016), an issue shared by many GCMs (Cesana et al., 2012; Forbes & Ahlgrimm, 2014). The LCCs that do occur in CAM5 produce snow too often relative to the observed frequency (McIlhattan et al., 2017). Downwelling longwave (LW) radiation at the surface is strongly connected with cloud presence and phase, thus is too low relative to both satellite derived Arctic estimates and measurements from a ground‐based observatory on Greenland (McIlhattan et al., 2017). Also related to the insufficient LCCs, Kay, Bourdages et al. (2016) found a summertime cold bias of 2–3°C in CAM5's daily maximum of near‐surface air temperatures at Summit, Greenland. In light of these known issues, improving Arctic cloud liquid representation was a goal for CAM6.

Studies comparing CESM1 with CAM5 and CESM2 with CAM6 (hereafter CESM1 and CESM2) have shown promising results in a variety of areas. The changes to CAM6 have increased correlation between model global monthly means and observations for a variety of atmospheric variables including: shortwave (SW) and LW cloud radiative effect; 30°S–30°N rainfall; and temperature (Gettelman et al., 2019). Northern Hemisphere circulation characteristics have improved, especially winter jet streams, storm tracks, and stationary waves (Simpson et al., 2020). Radiation biases over the southern ocean were reduced in development versions of CAM6 due to increased supercooled liquid in low‐level clouds (Bogenschutz et al., 2018). Lenaerts et al. (2020) found that overall CAM6 has improved cloud representation over the GrIS; in particular, LCCs are simulated in CAM6 in similar distributions to observations, whereas CAM5 simulates no LCCs over the GrIS outside coastal regions.

While CAM6 simulates some aspects of the atmospheric conditions over the GrIS well, that does not necessarily mean the atmosphere over the remaining land surfaces, sea ice, or open ocean will be well represented. Nor do improved global means guarantee Arctic improvement. In this work, we explore differences between CESM1 and CESM2 in cloud behavior over the whole Arctic. In order to see changes in the mean state rather than responses to transient forcing, we use fully coupled preindustrial control runs from the two versions. CESM2 historical and present‐day forcings are consistent with CMIP6 specifications, which are changed from the CMIP5 forcings used in CESM1—so changes in the cloud fields in simulations of the present‐day could be due to both forcing differences and model physics differences. By comparing 1850s preindustrial control runs averaged over many years, we can isolate the differences in the model representation of physical processes.

In the following sections, we aim to answer the questions:

- How has the frequency of Arctic LCCs changed in CESM2 relative to CESM1?
- How does the surface energy balance of the Arctic compare in the two versions?
- How has precipitation changed moving to CESM2, both in amount and spatial and temporal distribution?

The goal of this work is to document differences in the simulated Arctic climate between CESM1 and CESM2. We include observational data in some plots to provide context for particular variables. Our analysis illustrates how the combined updates to model parameterizations and physics in CESM2 have altered the representation of Arctic clouds, precipitation, and the broader Arctic climate.

## 2 Methods

### 2.1 Community Earth System Model

Our focus is to compare the overall cloud representation of CESM1 and CESM2, so we first compare the basic state of clouds and precipitation from multicentury, stable, preindustrial (1850s forcings) control runs for each version. For standard CESM variables, we use years 400–1199. In order to examine cloud properties that are not standard outputs for CESM, we have run fully coupled 10‐year branch simulations off of the control runs for both CESM1 and CESM2 with the necessary additional outputs but no change in configuration. The 10‐year branch simulations capture the annual mean and seasonal cycle of the long‐running control simulations (see Supporting Information S1). The additional variables output in the branch simulations are described in Table 1. All model outputs have a horizontal resolution of 1.25° longitude and ∼0.94° latitude. The Arctic is defined as all CESM grid boxes fully above the Arctic Circle, which encompasses the area of ∼66.91–90°N.

**Table 1:** Summary of High‐Frequency and Tendency Term CESM Variables Used in This Analysis

Variable	Description	Native units	Output type
“TGCLDLWP”	Total grid‐box cloud liquid water path	kg m^−2^	6‐hourly instantaneous
“TGCLDIWP”	Total grid‐box cloud ice water path	kg m^−2^	6‐hourly instantaneous
“PRECT”	Total (convective and large‐scale) precipitation rate (liq + ice)	m s^−1^	6‐hourly instantaneous
			
“VDCLDLIQ”	Vertical diffusion of CLDLIQ	kg kg^−1^ s^−1^	Monthly mean
“DCCLDLIQ”	CLDLIQ tendency due to moist processes	kg kg^−1^ s^−1^	Monthly mean
			
“MPDLIQ”	CLDLIQ tendency (Morrison microphysics)	kg kg^−1^ s^−1^	Monthly mean
			
“MACPDLIQ” (only CESM1)	CLDLIQ tendency from revised macrophysics	kg kg^−1^ s^−1^	Monthly mean
			
“SHDLFLIQ” (only CESM1)	Detrained liquid water from shallow convection	kg kg^−1^ s^−1^	Monthly mean
			
“CMFDLIQ” (only CESM1)	CLDLIQ tendency from	kg kg^−1^ s^−1^	Monthly mean
	shallow convection		
“ZMDLIQ”	CLDLIQ tendency from Zhang‐McFarlane convection	kg kg^−1^ s^−1^	Monthly mean
			
“DPDLFLIQ”	Detrained liquid water from deep convection	kg kg^−1^ s^−1^	Monthly mean
			
“RCMTEND_CLUBB” (only CESM2)	CLDLIQ tendency from CLUBB physics	kg kg^−1^ s^−1^	Monthly mean
			
“MPDW2P”	Water <–> Precip tendency (Morrison microphysics)	kg kg^−1^ s^−1^	Monthly mean
			
“MPDW2I”	Water <–> Ice tendency (Morrison microphysics)	kg kg^−1^ s^−1^	Monthly mean
			
“MPDW2V”	Water <–> Vapor tendency (Morrison microphysics)	kg kg^−1^ s^−1^	Monthly mean
			
“QCSEDTEN”	Cloud water mixing ratio tendency from sedimentation	kg kg^−1^ s^−1^	Monthly mean
			
“PRCO”	Autoconversion of cloud water	kg kg^−1^ s^−1^	Monthly mean
“PRAO”	Accretion of cloud water by rain	kg kg^−1^ s^−1^	Monthly mean
“PSACWSO”	Accretion of cloud water by snow	kg kg^−1^ s^−1^	Monthly mean
“BERGSO”	Conversion of cloud water to snow from Bergeron	kg kg^−1^ s^−1^	Monthly mean
			

*Note*. Variables were output from both CESM versions unless otherwise noted.

#### 2.1.1 Community Earth System Model Version 1

Both the CESM1 preindustrial control and branch run use the configuration from the CESM Large Ensemble project which is described in detail in Kay et al. (2015). Components included in CESM1 are as follows: atmosphere (CAM5), ocean (Parallel Ocean Program [POP], version 2), land (Community Land Model [CLM], version 4), sea ice (Los Alamos Sea Ice Model [CICE], version 4), land ice (Community Ice Sheet Model [CISM], version 1.9), and river (River Transport Model [RTM]). CESM1 modern era values (years 2007–10) are obtained from the original 30 ensemble members of the CESM Large Ensemble project (Kay et al., 2015).

#### 2.1.2 Community Earth System Model Version 2

Both the CESM2 preindustrial control and branch run use the configuration described in Danabasoglu et al. (2020). The components included are as follows: atmosphere (CAM6), ocean (POP version 2, with physical improvements), land (CLM version 5), sea ice (CICE version 5.1.2), land ice (CISM version 2.1), river (Model for Scale Adaptive River Transport [MOSART]), and wave (OAA WaveWatch‐III ocean surface wave prediction model [WW3]). CESM2 modern era values (years 2007–10) are obtained from the original 11 ensemble members run with CAM6 as part of the CESM2 release (Danabasoglu et al., 2020).

### 2.2 Satellite Observations

While this paper is focused on changes from one model version to another, it is helpful to anchor particular model variables with observed values to provide context. Details on how the observational data set is compared to model output are presented in section 2.3.

The observations leveraged in this work are derived from two instruments in the National Aeronautics and Space Administration A‐Train satellite constellation: *CloudSat*'s 94‐GHz Cloud Profiling Radar (CPR) and *CALIPSO*'s Cloud Aerosol Lidar with Orthogonal Polarization (CALIOP) (532‐ and 1,064‐nm wavelengths). Together, these active sensors have provided vertical column information in the Earth's atmosphere between 82°S and 82°N since 2006 (L'Ecuyer & Jiang, 2010). Their combined skill allows for both the determination of Arctic cloud phase as well as precipitation characteristics below the cloud layer (Battaglia & Delanoë, 2013). The particular data set used here was developed and detailed in McIlhattan et al. (2017) utilizing R04 versions of CloudSat Data Processing Center data products and boundaries of 66.91°N and 81.99°N for the Arctic. A satellite footprint is defined as containing an LCC if the nearest surface cloud layer in 2B‐CLDCLASS‐LIDAR is flagged liquid or mixed‐phase. An LCC is defined as precipitating if 2C‐PRECIP‐COLUMN contains any of the following flags: Snow Certain, Mixed Certain, Rain Certain, and Rain Probable. The broadband surface radiation fluxes are from 2B‐FLXHR‐lidar, an algorithm which utilizes cloud and precipitation property measurements from CloudSat and CALIPSO, along with temperature and humidity profiles from reanalyses, to initialize a radiative transfer model. Satellite footprints from January 2007 to December 2010 were gridded to the CESM resolution before taking the area‐weighted averages used in this work. We exclude data after 2010 because CloudSat experienced a battery malfunction in 2011 and has since only collected daytime data. For specific details on the satellites, data products, and validation, refer to the methods section in McIlhattan et al. (2017).

### 2.3 Comparing Model Output and Observational Data

The primary purpose of this paper is to document mean state changes in Arctic clouds and precipitation between CESM1 and CESM2. It is, nevertheless, useful to include observational values in some of our model comparisons to provide an independent frame of reference for the results.

The model results shown in tables and marked with solid lines in plots are area‐weighted averages of the full Arctic region (∼66.91–90°N). When comparing to observations, the model area defined as the Arctic is reduced to grid boxes between 66.91°N and 81.99°N to match the spatial extent of satellite observations described in section 2.2. In the plots, the reduced area model averages are shown with dashed lines.

When looking at clouds, model output and observations cannot be directly compared. Both scale and sensitivity must be taken into account before a meaningful comparison can be made (Kay et al., 2018). Clouds and their microphysical processes are parameterized in GCMs because they occur on scales far smaller than a single grid box. How a cloud is defined in an observational data set depends on the sensitivity of the instrument used. Satellite simulators have been developed to bridge the gap between modeled and observed clouds (Kay et al., 2012); however, the satellite data used in this work are a combination of radar and lidar that is not currently available as a simulator package. To compare LCC frequency (equivalent to cloud fraction) and LCC precipitation frequency between CESM and CloudSat/CALIPSO observations, we use the thresholds developed in McIlhattan et al. (2017). Specifically, model LCCs are defined as grid boxes containing 6‐hourly instantaneous values ≥5 g m^−2^ of vertically integrated cloud liquid (“TGCLDLWP”), and model LCCs are defined as precipitating if they have 6‐hourly instantaneous precipitation values (“PRECT”) ≥0.01 mm hr^−1^. If the frequency of LCCs is below 2% in a given month, that month is masked when calculating precipitation frequency to avoid a sampling bias. In developing these thresholds, McIlhattan et al. (2017) attempted to mimic the detection limits of the satellite instruments, though the scale difference could not be accounted for with a simple threshold. The scale mismatch likely results in the model LCC frequency biased low in places that are slightly below the threshold and biased high in areas slightly above the threshold. However, a sensitivity test showed that for CESM1, the results were robust and not dependent on the exact threshold adopted (McIlhattan et al., 2017, Fig. 5). The threshold of ≥5 g m^−2^ captures a large fraction of the radiatively important LCCs. Bennartz et al. (2013) used an energy balance model to examine the radiative impact of LCCs over the GrIS and found liquid water paths below 10 g m^−2^ were not opaque enough to trap LW radiation efficiently.

It is important to remember that the model output for both CESM1 and CESM2 is from preindustrial simulations, while the satellite data are from the modern era (2007–10). Since sea ice extent, greenhouse gas levels, surface temperatures, and other atmospheric variables are different in the modern era than they were in the 1850s, an exact match between the satellite and observations is neither expected or desired. In evaluating the mean state differences between CESM1 and CESM2, we chose not to compare modern era simulations because that would convolute both mean state and transient forcing changes. Bogenschutz et al. (2018) used a similar method to what we present here, using modern era observational data sets to frame preindustrial control changes between intermediate CAM6 versions. Such comparisons are meaningful because often times differences between present‐day and preindustrial are much smaller than differences between model and observations (discussed further in section 3.1). Where available and appropriate, satellite observations are included to provide a present‐day reference for the physically reasonable range of particular variables.

## 3 Results

### 3.1 Cloud Representation

**Figure 1:**
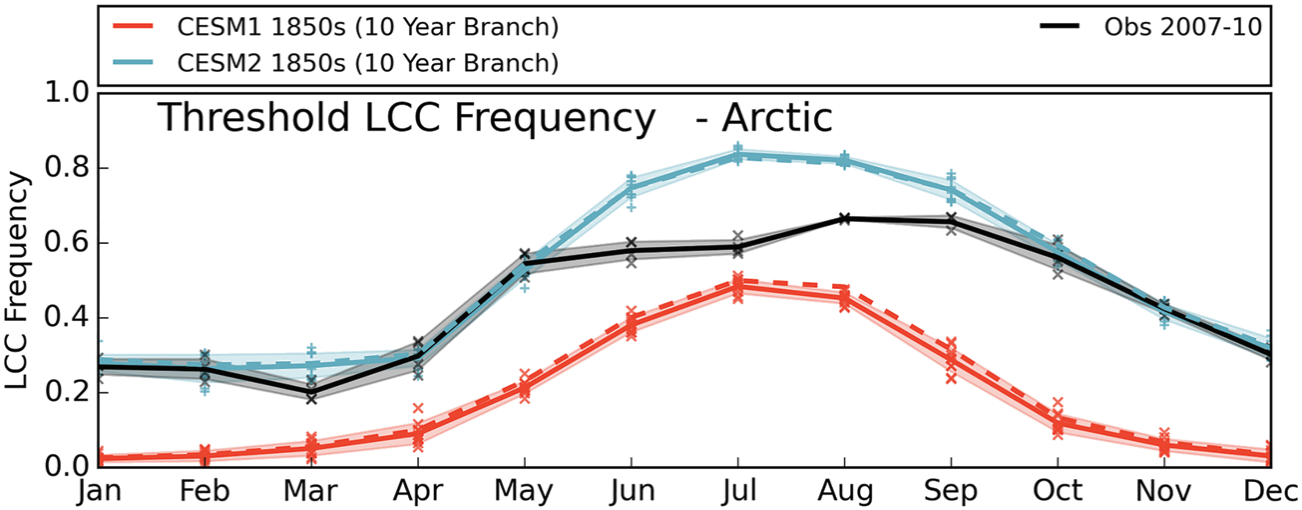
Annual cycle of liquid‐containing cloud (LCC) frequency in the Arctic. The solid lines for CESM1 (red) and CESM2 (blue) illustrate the mean values for the monthly area‐weighted averages for all grid boxes between 66.91°N and 90°N from the 10‐year branch simulations of their respective preindustrial control runs. The dashed lines are also for CESM1 and CESM2, but for the area between 66.91°N and 81.99°N, for comparison with observations. The black line represents the average of 2007–10 CloudSat/CALIPSO observations. The markers surrounding the solid lines each depict a single year's monthly average. The shaded regions denote the standard deviation about the mean for the month, showing the interannual variability.

Clouds influence the Arctic surface energy balance, modulating the radiation received at the surface. LCCs in particular have a large impact on downwelling LW radiation (Van Tricht et al., 2016) and are ubiquitous throughout the Arctic (H.  Morrison et al., 2012). Too few Arctic LCCs is a known and documented issue for CESM1 (Cesana et al., 2015; Kay, Bourdages et al., 2016; McIlhattan et al., 2017; Tan & Storelvmo, 2016). To assess Arctic LCC representation in CESM1 and CESM2, the frequency of Arctic LCCs (6‐hourly instantaneous values ≥5 g m^−2^ “TGCLDLWP” divided by total number of instantaneous values) is shown in Figure 1. Most line plots follow the same format: monthly mean model results for the full Arctic are depicted by a solid line, while results for the reduced satellite observation extent are depicted by a dashed line; individual monthly values are depicted by markers; the shaded regions are the standard deviation about the mean; and the data source (control run or branch simulation) is noted in the figure caption. In all months, the frequency of Arctic LCCs in CESM2 (blue) is increased dramatically relative to CESM1 (red). The annual cycles for the two model versions show a similar shape, with LCCs at a minimum in winter and maximum in summer, peaking in July.

We have included modern era (2007–10) observed values (black line) in Figure 1 to show one physically reasonable annual cycle for Arctic LCCs. The 66.91–81.99°N area‐weighted model means (dashed red and blue lines in Figure 1) are qualitatively the same as the full Arctic (66.91–90°N) model values for LCC frequency, so will not be separately discussed. The CESM2 1850s branch run is similar in magnitude to modern era observations in all months except June–September. In those summer and early fall months, CESM2 contains more Arctic LCCs than observed. In fall, open ocean and warmer temperatures favor Arctic LCCs (A. L.  Morrison et al., 2019), so we would expect the fall modern era, with its increased temperatures and decreased sea ice, to have more LCCs relative to the fall 1850s. Since 1850s CESM2 has more fall LCCs than are observed in the modern era, it may now be overestimating relative to what is physically reasonable. This potential overestimation is also shown by the annual mean values (Table 2), with CESM2 having more than twice the LCC frequency of CESM1 (0.52 and 0.20, respectively for the reduced observational area) and higher than the observed value (0.45).

**Table 2:** Summary of Annual Mean Values of Arctic Cloud Properties

Data Set	Spatial region	Time period	LCC frequency	Total cloud water (kg m^−2^)	Cloud liquid water (kg m^−2^)	Cloud ice water (kg m^−2^)
CESM1	67–90°N	1850s branch	0.19 ± 0.01	—	—	—
CESM1	67–90°N	1850s control	—	0.0205 ± 0.0009	0.0122 ± 0.0007	0.0083 ± 0.0005
CESM1	67–90°N	2007–10	—	0.0246 ± 0.0010	0.0158 ± 0.0008	0.0087 ± 0.0005
CESM2	67–90°N	1850s branch	0.51 ± 0.01	—	—	—
CESM2	67–90°N	1850s control	—	0.0739 ± 0.0022	0.0682 ± 0.0022	0.0057 ± 0.0003
CESM2	67–90°N	2007–10	—	0.0945 ± 0.0024	0.0883 ± 0.0025	0.0062 ± 0.0004
CESM1	67–82°N	1850s branch	0.20 ± 0.01	—	—	—
CESM2	67–82°N	1850s Branch	0.52 ± 0.01	—	—	—
Obs.	67–82°N	2007–10	0.45 ± 0.02	—	—	—

*Note*. The ± value is the standard deviation of the annual mean values. The “1850s Control” is composed of 800 years of the preindustrial control simulation. The “1850s Branch” is the 10‐year branch simulation run from the preindustrial control. The “2007–10” time period for the model years 2007–10 of the historical ensemble members (30 members for CESM1 and 11 members for CESM2). The top section contains area‐weighted means for the full modeled Arctic (66.91–90°N), while the bottom section contains the area‐weighted means for the observed Arctic (66.91–81.99°N).

**Figure 2:**
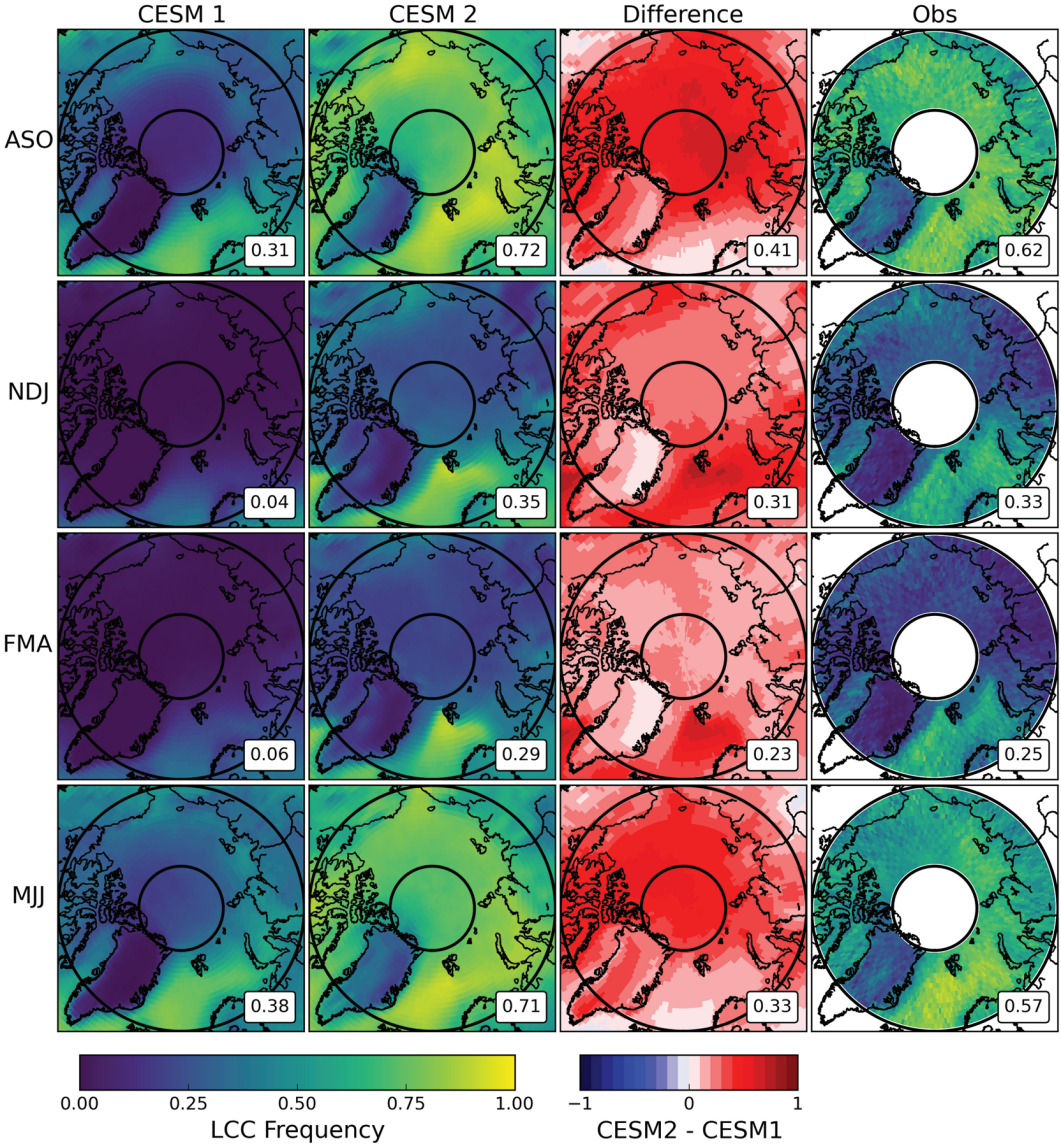
Seasonal averages of LCC frequency in the Arctic. Seasonal divisions were chosen to capture sea ice minimum (August, September, and October, top row) and sea ice maximum (February, March, and April, third row). The plots for CESM1 (first column) and CESM2 (second column) are means from the 10‐year branch simulations off their respective 1850s control runs. The difference plots in the third column are CESM2 minus CESM1, with red (blue) values showing increases (decreases) in LCC frequency in CESM2 with respect to CESM1. The fourth column are observational values from CloudSat/CALIPSO. The area‐weighted averages for the observational area (66.81°N and 81.99°N) are shown in the lower right of each map.

The seasonal and spatial distribution of Arctic LCC frequency is shown in Figure 2. The area‐weighted mean seasonal values in the bottom right of all maps correspond to the observational area 66.91–81.99°N. CESM1 and CESM2 have qualitatively similar distributions of LCC in each season (Figure 2, first and second columns). For winter (NDJ) and spring (FMA), CESM1 produces LCCs very infrequently (0.04 and 0.06, respectively) and almost exclusively in the North Atlantic region. Winter and spring LCCs in CESM2 are also concentrated in the North Atlantic but appear in moderate numbers in the Chukchi Sea and small numbers throughout the Arctic. These changes are illustrated in the difference plots (Figure 2, third column) with the largest winter and spring increases highlighting the North Atlantic and Chukchi Sea and the smallest increases over the GrIS. CESM1 produces more LCCs in summer (MJJ) and fall (ASO) (0.38 and 0.31, respectively) than the colder seasons, save over the GrIS where no LCCs occur outside the coastline. As we saw in Figure 1, the summer and fall maps show the largest magnitude increases in CESM2 LCC frequency relative to CESM1 (+0.33 and +0.41, respectively). The differences in the warmer seasons are large everywhere except the North Atlantic, where values were already high in CESM1. Maps of the observed modern‐day LCC frequency (Figure 2, fourth column) are qualitatively quite similar to CESM2 across all four seasons, indicating that the increases in CESM2 LCCs have resulted in a spatial distribution consistent with the real world.

**Figure 3:**
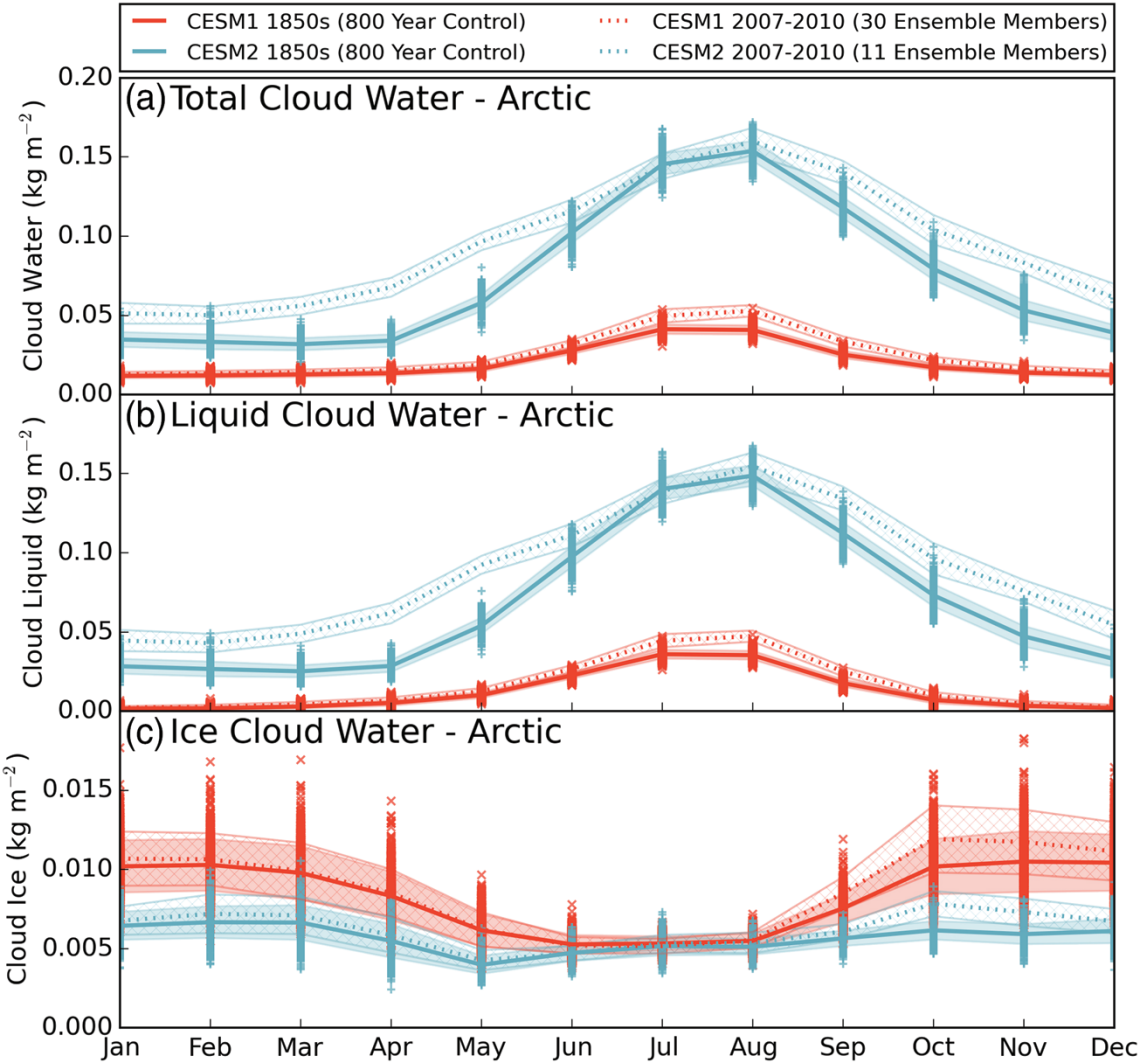
As in Figure 1 for the annual cycles of (a) total cloud water, (b) liquid cloud water, and (c) ice cloud water. Note the same*y*‐axis is used in (a) and (b), but (c) is reduced. Model data are from years 400–1199 of the preindustrial control runs (solid lines) and from years 2007–10 of the historical ensemble members (dotted lines, 30 members for CESM1 and 11 members for CESM2).

Does this increase in LCC frequency indicate an overall increase in Arctic cloudiness, or does it come at the expense of ice clouds? To explore this question, we look at the annual cycle of total cloud water path (monthly mean “TGCLDLWP” + “TGCLDIWP,” Figure 3a). Preindustrial Arctic total cloud water (liquid + ice, solid lines) is higher in CESM2 than CESM1 in all months. The two model versions have similarly shaped annual cycles, with relatively constant values from December–April and a peak around July and August. However, the absolute amount of Arctic cloud water in CESM2 is more than doubled in the winter months and more than quadrupled in September and October, relative to CESM1 values. The annual mean value has more than tripled, going from 0.0205 kg m^−2^ in CESM1 to 0.0739 kg m^−2^ in CESM2 (Table 2). Dividing the total water by phase, we see that the annual cycles of liquid cloud water (monthly mean “TGCLDLWP,” Figure 3b) for both CESM1 and CESM2 are similar to the total cloud water, showing much more liquid in the new model version. In the annual average, the Arctic cloud liquid water in CESM2 (0.0682 kg m^−2^) is more than five times larger than in CESM1 (0.0122 kg m^−2^). Cloud ice water (monthly mean “TGCLDIWP,” Figure 3c), on the other hand, is decreased in CESM2 relative to CESM1, but noting the reduced value of the *y*‐axis for cloud ice, the absolute value of the change is smaller for cloud ice than cloud water. Indeed, there is reduced cloud ice and increased cloud water in CESM2, but it is apparent that the increase in cloud liquid is due to an overall increase in Arctic cloud water, not simply a transition from cloud ice to cloud liquid.

Modern era values for cloud water (years 2007–10, dotted lines) are included in Figure 3 and Table 2 to highlight the relative change in Arctic cloud water between model versions and between time periods. Going from the preindustrial to the modern era, CESM1 has a slight increase in summertime liquid cloud water (Figure 3b) and a slight increase in fall and winter cloud ice water (Figure 3c). CESM2 displays a larger dependence on time period; in all months except July and August, the modern era Arctic cloud liquid is well above the standard deviation of the preindustrial values (Figure 3b, blue shaded region). However, for the cloud ice water, the CESM2 modern era increase is small, similar to that of CESM1 (Figure 3c). In the annual average, the difference between the preindustrial and modern era Arctic cloud liquid is +0.0036 kg m^−2^ for CESM1 and +0.0201 kg m^−2^ for CESM2 (Table 2), which is a ∼30% increase for both versions. For Arctic cloud ice, that difference is +0.0004 kg m^−2^ for CESM1 and +0.0005 kg m^−2^ for CESM2, a <10% increase for both versions. While the model values do increase between the preindustrial and the modern‐day for each version, as expected with a warming climate, it is the change between the two model versions that has a larger impact on preindustrial Arctic cloud water, both in magnitude (+0.053 kg m^−2^ for cloud liquid and −0.0026 kg m^−2^ for cloud ice) and percent change (+260% for cloud liquid and −31% for cloud ice). The relatively small time period changes for cloud content increases confidence in the comparisons made between modern era satellite observations and preindustrial model simulations. Only preindustrial values will be shown and discussed the in the remainder of the results.

**Figure 4:**
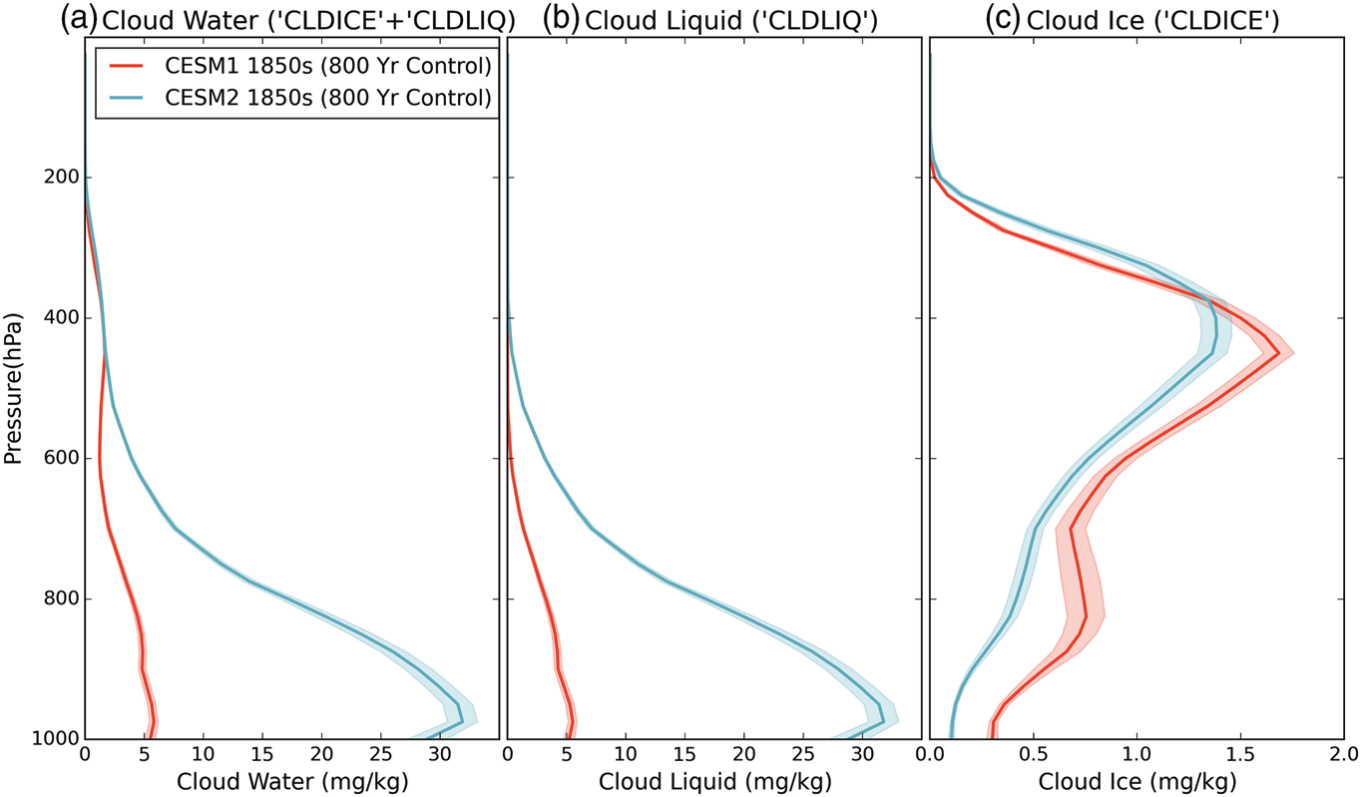
Annual average vertical profiles for (a) total cloud water, (b) liquid cloud water, and (c) ice cloud water. Note the same*x*‐axis is used in (a) and (b), but (c) is reduced. The shaded regions denote the standard deviation about the mean for a given pressure level. Model data are from years 400–1199 of the preindustrial control runs.

An examination of the distribution of Arctic cloud water in the vertical column (monthly mean “CLDLIQ” + “CLDICE,” Figure 4a) reveals that the largest increase in CESM2 cloud water occurs near the surface, below 900 hPa. As we saw in the annual cycle of cloud water, the difference between the two versions is dominated by the change in cloud liquid (monthly mean “CLDLIQ,” Figure 4b). The vertical profile of cloud ice (monthly mean “CLDICE,” Figure 4c) shows that there is a slight increase in CESM2 cloud ice relative to CESM1 above the 375‐hPa level and a decrease everywhere between the surface and that pressure level. However, the cloud liquid change is an order of magnitude larger than the cloud ice change, and the main difference in the CESM2 vertical profile relative to CESM1 is an increase of cloud liquid at low levels.

### 3.2 Surface Radiative Fluxes and Temperature

With large changes in clouds, it is certainly interesting and important to also assess changes in radiation. In particular, with the substantial increase in Arctic cloud liquid in CESM2, we expect increased downwelling LW radiation due to enhanced trapping of terrestrial radiation and decreased downwelling SW radiation at the surface due to enhanced reflection of incoming solar radiation. Since the LW and SW radiation changes have competing effects, the total downwelling radiation as well as the surface and near surface temperatures could increase or decrease depending on which effect dominates.

#### 3.2.1 Downwelling Radiation at the Surface

**Figure 5:**
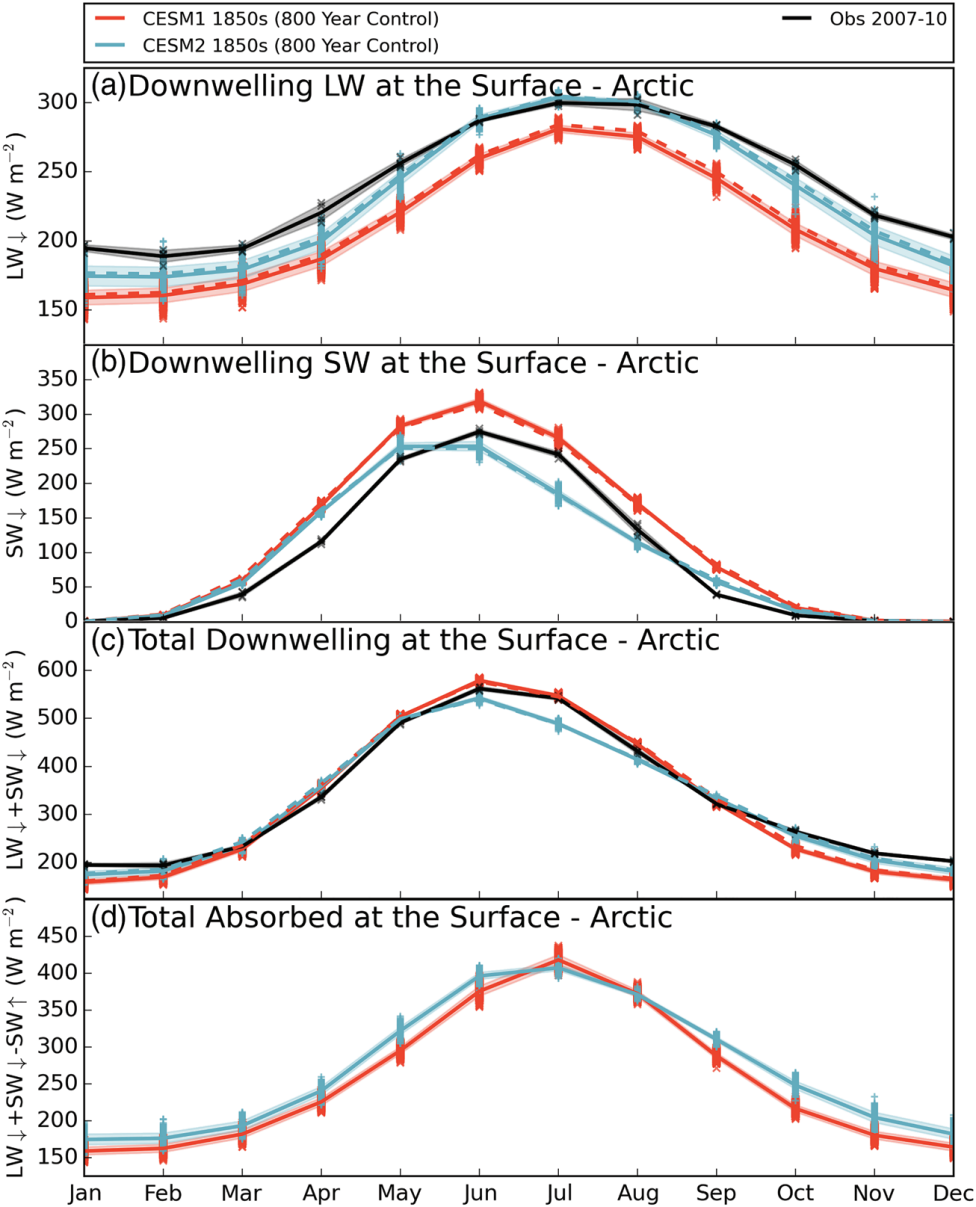
As in Figure 1 for the annual cycles of (a) downwelling LW radiation at the surface, (b) downwelling SW radiation at the surface, (c) total downwelling radiation at the surface (LW*↓* + SW*↓*), and (d) total absorbed radiation at the surface(LW*↓* + SW*↓* − SW*↑*). Note each *y*‐axis is different. Model data are from years 400–1199 of the preindustrial control runs. The observationally constrained surface fluxes are from the CloudSat/CALIPSO data product 2B‐FLXHR‐lidar.

Looking first at the changes in downwelling LW radiation (monthly mean “FLDS,” Figure 5a), we see that CESM2 has consistently larger values year round relative to CESM1 but maintains the same annual cycle shape. The annual means in Table 3 show that CESM2 has on average 22 W m^−2^ more downwelling LW radiation at the surface than CESM1. The modern era observed values follow a similar annual cycle but with larger values than both CESM1 and CESM2 in all months except June, July, and August where observations overlap CESM2 LW values. The peak values occur in July and August in all three data sets, which coincides with the peak in liquid cloud water discussed in section 3.1.

**Table 3:** Summary of Annual Mean Values for Downwelling Surface Radiation in the Arctic

Data set	Spatial region	Time period	LW↓ (W m^−2^)	SW↓ (W m^−2^)	Total↓ (LW↓ + SW↓) (W m^−2^)	Total absorbed (LW↓ + SW↓ − SW↑) (W m^−2^)
CESM1	67–90°N	1850s control	209 ± 2	115 ± 1	325 ± 2	254 ± 3
CESM2	67–90°N	1850s control	231 ± 3	92 ± 1	323 ± 2	270 ± 3
CESM1	67–82°N	1850s control	212 ± 2	116 ± 1	328 ± 2	—
CESM2	67–82°N	1850s control	233 ± 2	93 ± 1	326 ± 2	—
Obs.	67–82°N	2007–10	242 ± 3	95 ± 4	337 ± 5	—

*Note*. The ± value is the standard deviation of the annual values. The top section contains area‐weighted means for the full modeled Arctic (66.91–90°N), and the bottom section contains the area‐weighted means for the observed Arctic (66.91–81.99°N). Model data are from years 400–1199 of the preindustrial control runs. The observationally constrained surface fluxes are from the CloudSat/CALIPSO data product 2B‐FLXHR‐lidar.

The increase in cloud water in CESM2 has also impacted the SW reaching the Arctic surface (monthly mean “FSDS,” Figure 5b). Not only is the magnitude of the downwelling SW radiation reduced in CESM2 relative to CESM1, but the annual variation has also changed. The largest reductions in SW occur in June, July, and August (Figure 5b), the same months with the largest cloud liquid increases (Figure 3b). In CESM1, there is a clear peak in downwelling SW radiation in June, but in CESM2, that peak has shifted earlier and is centered around May and June. In this case, the observed values more closely match the annual cycle of CESM1, having a clear June maximum and a smooth distribution around that peak. However, the observed annual mean of downwelling SW radiation is closer to the reduced area mean for CESM2 than CESM1 (95, 93, and 116 W m^−2^, respectively, Table 3).

The annual mean total value of downwelling radiation (monthly mean “FLDS” + “FSDS”) in the modeled Arctic is 2 W m^−2^ less in CESM2 than CESM1 (323 and 325 W m^−2^, respectively, Table 3). This is a relatively small change overall, indicating that the competing effects of SW and LW are nearly balanced. However, looking at the annual cycle of total downwelling radiation (Figure 5c), we can see that there are larger changes in individual seasons. In the late fall and winter (October–February), there is consistently more total radiation received at the surface in CESM2 relative to CESM1, consistent with the more frequent LCCs trapping more LW terrestrial radiation with no competing SW effect. Conversely, in the summer (June–August), CESM2 sees less total radiation at the surface than CESM1 owing in part to the increased cloud albedo from the greatly increased liquid water content. The observed values follow CESM1 closely in the spring, summer, and fall, and in winter, the total downwelling radiation is higher than both CESM1 and CESM2. The total radiation mismatch between CESM2 and observations in June, July, and August, when taken with overabundance of LCCs in those same months (Figure 1), indicates that CESM2 has possibly overcorrected and could be simulating too much cloud liquid relative to the real world. Even so, the differing time periods of the observations and models should be kept in mind and connections between the data sets not be overinterpreted.

**Figure 6:**
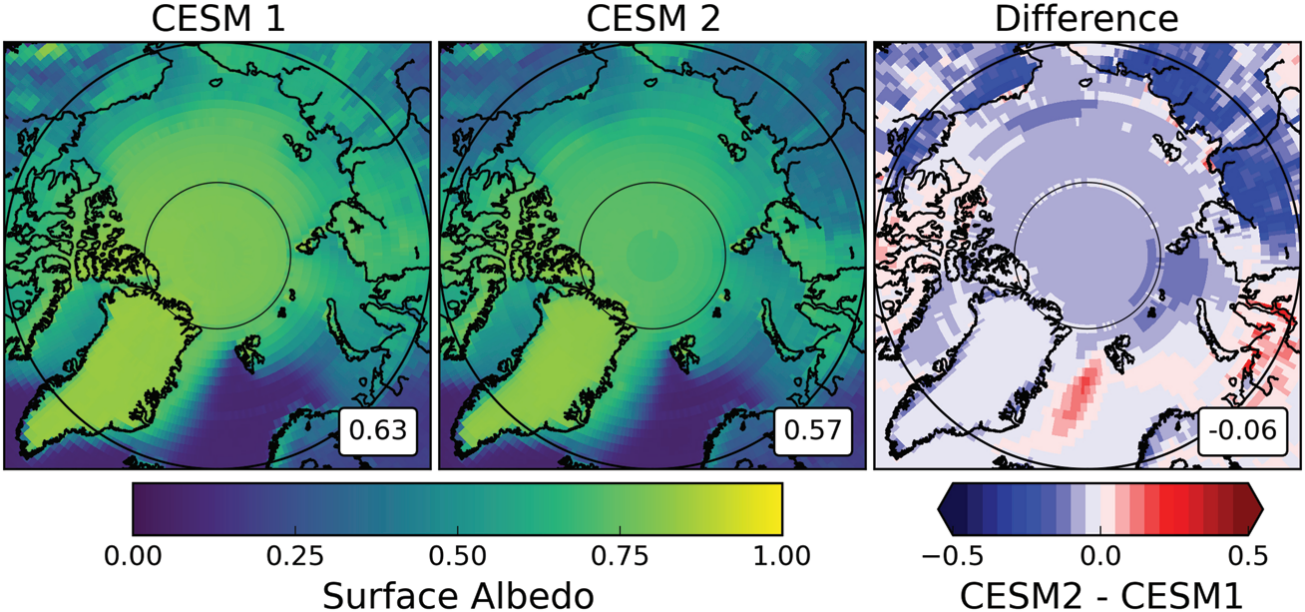
Annual averages of Arctic surface albedo. Calculated with model monthly means as follows: 1 − “FSNS”/“FSDS,” masked where “FSNS” ≤ 0.0001 W m^−2^. The plots for CESM1 (left) and CESM2 (center) are means from their respective 800‐year control runs. The difference plot on the right is CESM2 minus CESM1, with red (blue) values showing increases (decreases) in surface albedo in CESM2 with respect to CESM1. The area‐weighted averages for the study area (66.81°N and 90°N) are shown in the lower right of each map.

The amount of radiation absorbed by the Arctic surface is determined by both the total downwelling radiation (Figure 5c) and the emissivity of the surface. This work is not focused on Arctic surface changes between the two model versions; however, the annual cycle of total radiation absorbed at the surface (monthly mean “FLDS” + “FSNS,” Figure 5d) indicates that CESM2 absorbs more radiation at the surface in all months except July and August. Despite the annual total downwelling radiation decrease in CESM2 relative to CESM1 (−2 W m^−2^), the total absorbed at the surface has increased by 16 W m^−2^. This is at least partially due to the reduction in Arctic surface albedo (monthly means 1 − “FSNS”/“FSDS,” masked where “FSNS” ≤ 0.0001 W m^−2^) in CESM2 relative to CESM1 (57% and 63%, respectively), with spatial maps (Figure 6) indicating that both land and sea ice surfaces in CESM2 reflect less incoming SW.

#### 3.2.2 Surface Temperature

**Figure 7:**
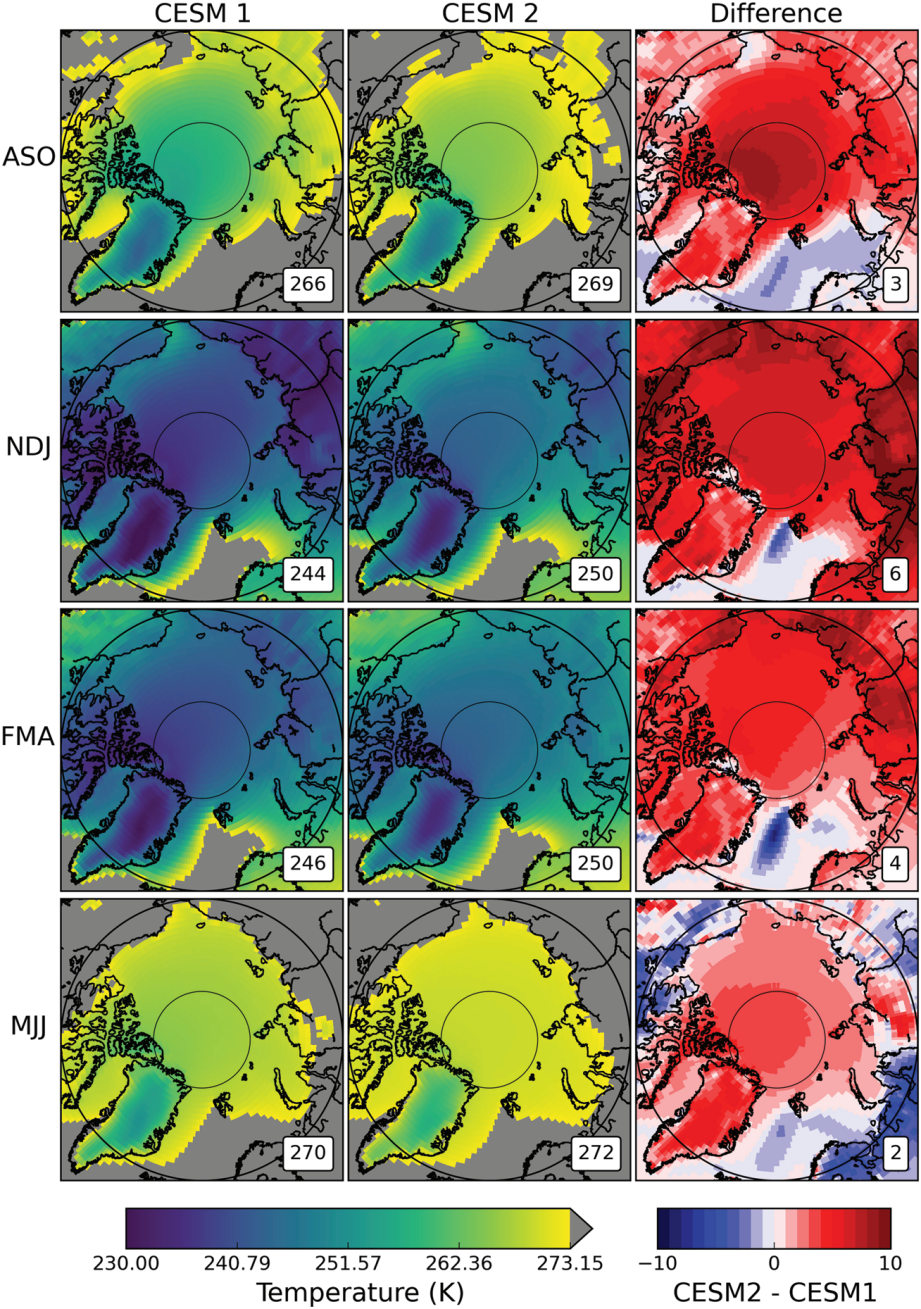
Seasonal averages of surface temperature in the Arctic. Seasonal divisions were chosen to capture sea ice minimum (August, September, and October, top row) and sea ice maximum (February, March, and April, third row). The plots for CESM1 (left column) and CESM2 (center column) are means from their respective 800‐year control runs. The difference plots in the right column are CESM2 minus CESM1, with red (blue) values showing increases (decreases) in temperature in CESM2 with respect to CESM1. The area‐weighted averages for the study area (66.81°N and 90°N) are shown in the lower right of each map.

While both the CESM1 and CESM2 data are 1850s control runs, it is clear that there are large differences in the mean cloud states which impact the surface radiation budget. The Arctic ocean, land, and ice surface temperatures are dependent on a variety of factors, of which downwelling radiation is only one (e.g., ocean circulation, sensible and latent heat fluxes, and emissivity). The spatial and seasonal variations of surface temperature (monthly mean “TS”) are shown in Figure 7. Values for near surface air temperature (“TREFHT”) are qualitatively similar to surface temperature in spatial distribution (not shown) and quantitatively similar in annual means (Table 4); thus, only surface temperature is discussed hereafter. Due to the warm ocean currents of the North Atlantic, both model versions have in all seasons a region of open water containing above‐freezing surface temperatures (depicted by solid grey). Likewise, in both model versions, the high and bright surfaces of the GrIS are consistently the coldest in every season. Nevertheless, there are large‐scale differences in the surface temperatures of the simulated Arctic between CESM1 and CESM2 (Figure 7, right column). CESM2 has a 3 K higher mean annual Arctic surface temperature than CESM1 (257 and 260 K, respectively, Table 4), but that difference is not distributed evenly spatially or temporally. The largest difference in mean surface temperature is in winter (NDJ), when the CESM2 Arctic is nearly 6 K warmer than the CESM1 Arctic. In winter, the increase in surface temperature is fairly evenly distributed across the Arctic, with the exception of the North Atlantic which is colder in CESM2 relative to CESM1. In summer and fall, when the largest increases in CESM2 LCCs occurred, we can see a concentration of warming over the sea ice at the north pole and the GrIS that somewhat resembles the pattern of increase in LCCs (Figure 2, third column). However, there are many factors that influence the Arctic surface temperature—surface albedo, ocean circulation, air mass temperature, etc.—and increased cloud liquid is only one of them. It should also be noted that the global temperature in CESM2 is higher than CESM1, though only by 1 K (Table 3). The modifications to CESM between version 1 and version 2 have undoubtedly altered the mean state of Earth's climate, including an overall warming that likely is partially due to changes to the clouds.

**Table 4:** Summary of Annual Mean Values for Surface and Near Surface Temperature in the Arctic

Data set	Spatial region	Time period	Surface temperature (K)	Near surface air temperature (K)
CESM1	67–90°N	1850s control	256.6 ± 0.6	257.0 ± 0.6
CESM2	67–90°N	1850s control	260.2 ± 0.6	260.2 ± 0.6
CESM1	Global	1850s control	287.1 ± 0.1	286.3 ± 0.1
CESM2	Global	1850s control	288.3 ± 0.1	287.2 ± 0.1
CESM1	67–82°N	1850s control	257.4 ± 1	257.8 ± 0.6
CESM2	67–82°N	1850s control	260.8 ± 1	260.8 ± 0.6
Obs.	67–82°N	2007–10	262.5 ± 0.6	262.8 ± 0.6

*Note*. The ± value is the standard deviation of the annual mean values. The top section contains area‐weighted means for the full modeled Arctic (66.91–90°N), the center section are global means, and the bottom section contains the area‐weighted means for the observed Arctic (66.91–81.99°N). Model data are from years 400–1199 of the preindustrial control runs. The observed temperature values are included in the CloudSat/CALIPSO data product 2B‐FLXHR‐lidar and are collocated temperature profiles for the satellite footprint provided by the European Centre for Medium‐Range Weather Forecasts (ECMWF) reanalysis product, AN‐ECMWF.

### 3.3 Precipitation

**Figure 8:**
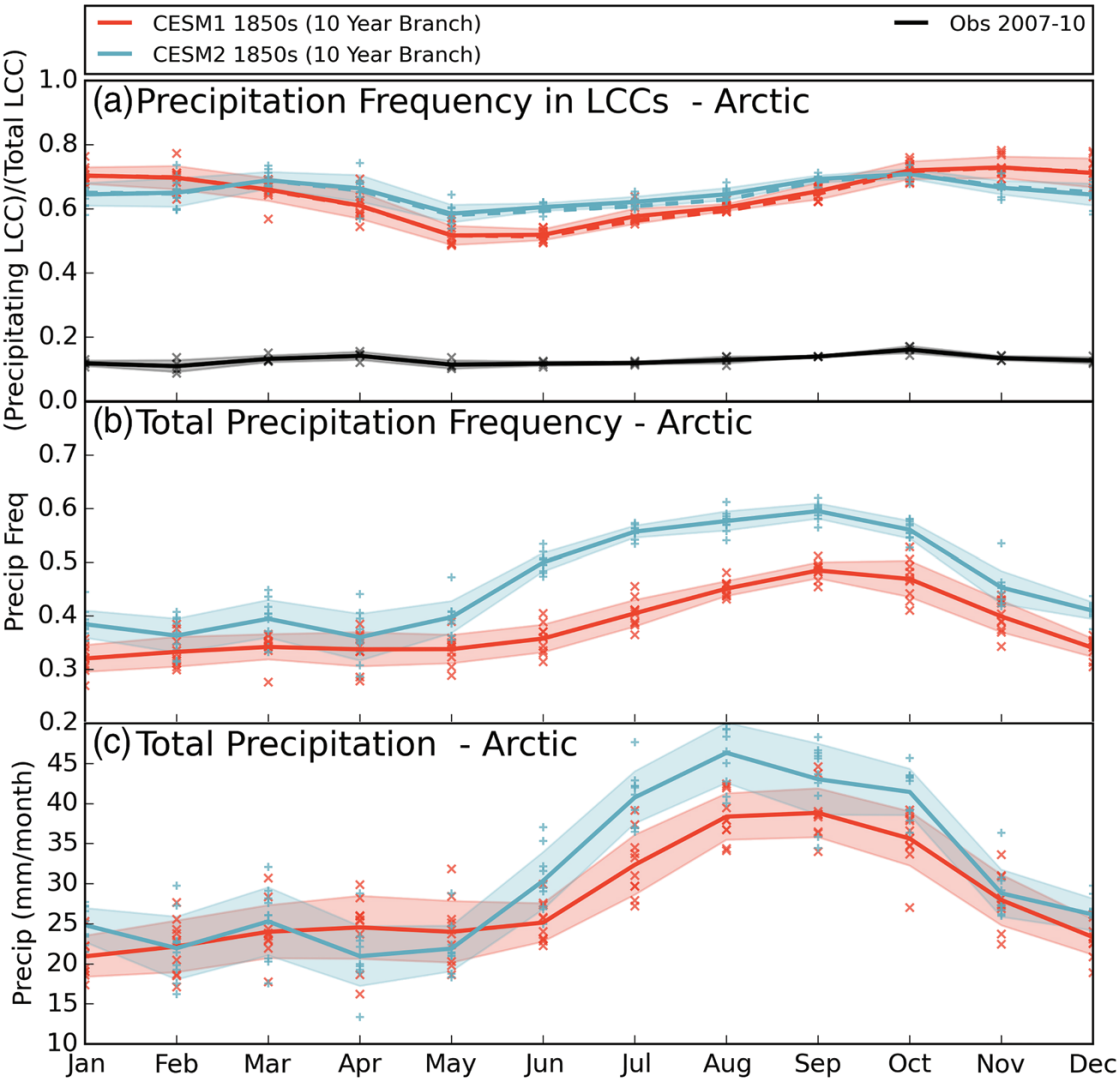
As in Figure 1 for the annual cycles of (a) precipitation frequency in LCCs, (b) total precipitation frequency, and (c) total precipitation. Note each*y*‐axis different. Model data are from the 10‐year branch simulations.

The large increase in simulated Arctic cloud liquid has implications for the Arctic water budget as well, since clouds govern when and where precipitation occurs. Previous work linked the dearth of CESM1 LCCs to too frequent snowfall (McIlhattan et al., 2017), which leads us to first compare how often this subset of clouds precipitates between the two versions. The annual cycle of precipitation frequency in LCCs (6‐hourly instantaneous values of both ≥5 g m^−2^ “TGCLDLWP” and ≥0.01 mm hr^−1^ “PRECT,” divided by total number of 6‐hourly instantaneous values of ≥5 g m^−2^ “TGCLDLWP”) is very similar between CESM2 and CESM1 (Figure 8a). The annual mean values in Table 5 show that both model versions have LCCs precipitating the majority of the time (64% and 65% of the time for CESM1 and CESM2, respectively). These values are sensitive to the thresholds we defined; however, the qualitative results are robust, and even with a much reduced LCC threshold (0.1 g m^−2^), modeled LCCs would still precipitate the majority of the time (not shown). Satellite observations, on the other hand, indicate that Arctic LCCs only produce precipitation 13% of the time (Table 5) with very little variability temporally (Figure 8a) or spatially (not shown). Ground‐based measurements from the GrIS have supported the satellite derived LCC precipitation frequency (McIlhattan et al., 2017). The consistency of the observationally based values possibly indicates a fundamental constraint on the lifetime of LCCs, which CESM has not yet been able to reproduce.

**Table 5:** Summary of Annual Mean Values for Arctic Precipitation

Data set	Spatial region	Time period	LCC precip frequency	Total precip frequency	Total precip rate (mm month^−1^)	Snow rate (mm month^−1^)	Rain rate (mm month^−1^)
CESM1	70–90°N	1850s branch	0.642 ± 0.009	0.382 ± 0.008	28 ± 1	—	—
CESM2	70–90°N	1850s branch	0.652 ± 0.006	0.463 ± 0.009	31 ± 1	—	—
CESM1	70–90°N	1850s control	—	—	—	20.2 ± 1.0	7.9 ± 0.7
CESM2	70–90°N	1850s control	—	—	—	18.8 ± 1.0	12.7 ± 0.8
CESM1	66.5–82°N	1850s branch	0.639 ± 0.008	—	—	—	
CESM2	66.5–82°N	1850s branch	0.648 ± 0.006	—	—	—	
Obs.	66.5–82°N	2007–10	0.129 ± 0.003	—	—	—	

*Note*. The ± value is the standard deviation of the annual mean values. The “1850s control” is composed of 800 years of the preindustrial control simulation. The “1850s branch” is the 10‐year branch simulation run from the preindustrial control. The top section contains area‐weighted means for the full modeled Arctic (66.91–90°N), and the bottom section contains the area‐weighted means for the observed Arctic (66.91–81.99°N).

Looking at total precipitation frequency (6‐hourly instantaneous values ≥0.01 mm hr^−1^ “PRECT”), Figure 8b shows that CESM2 has a higher mean precipitation frequency in all months compared to CESM1. In winter and spring, the models' monthly means are similar in magnitude, and there is overlap of the monthly variability (shaded regions), whereas in the summer and fall, CESM2 clearly precipitates much more often than CESM1 with no overlap in monthly variability. In the Arctic annual average, CESM2 precipitates 46% of the time, while CESM1 precipitates 38% of the time (Table 5).

While the precipitation frequency has seen a large increase in the updated model, the annual mean precipitation amount in the Arctic (6‐hourly instantaneous “PRECT”) is only slightly increased in CESM2 compared to CESM1 (31 and 28 mm month^−1^, respectively, Table 5). This means that not only is Arctic precipitation overall more frequent in CESM2 than CESM1, but it is also overall lighter. Comparing the annual cycles of frequency and amount (Figures 8b and 8c, respectively), we see that it is the late winter and spring in particular when CESM2 has more frequent precipitation but similar or less total precipitation relative to CESM1, indicating that the mean CESM2 precipitation event is even lighter in those seasons.

#### 3.3.1 Snowpack on Sea and Land Ice

**Figure 9:**
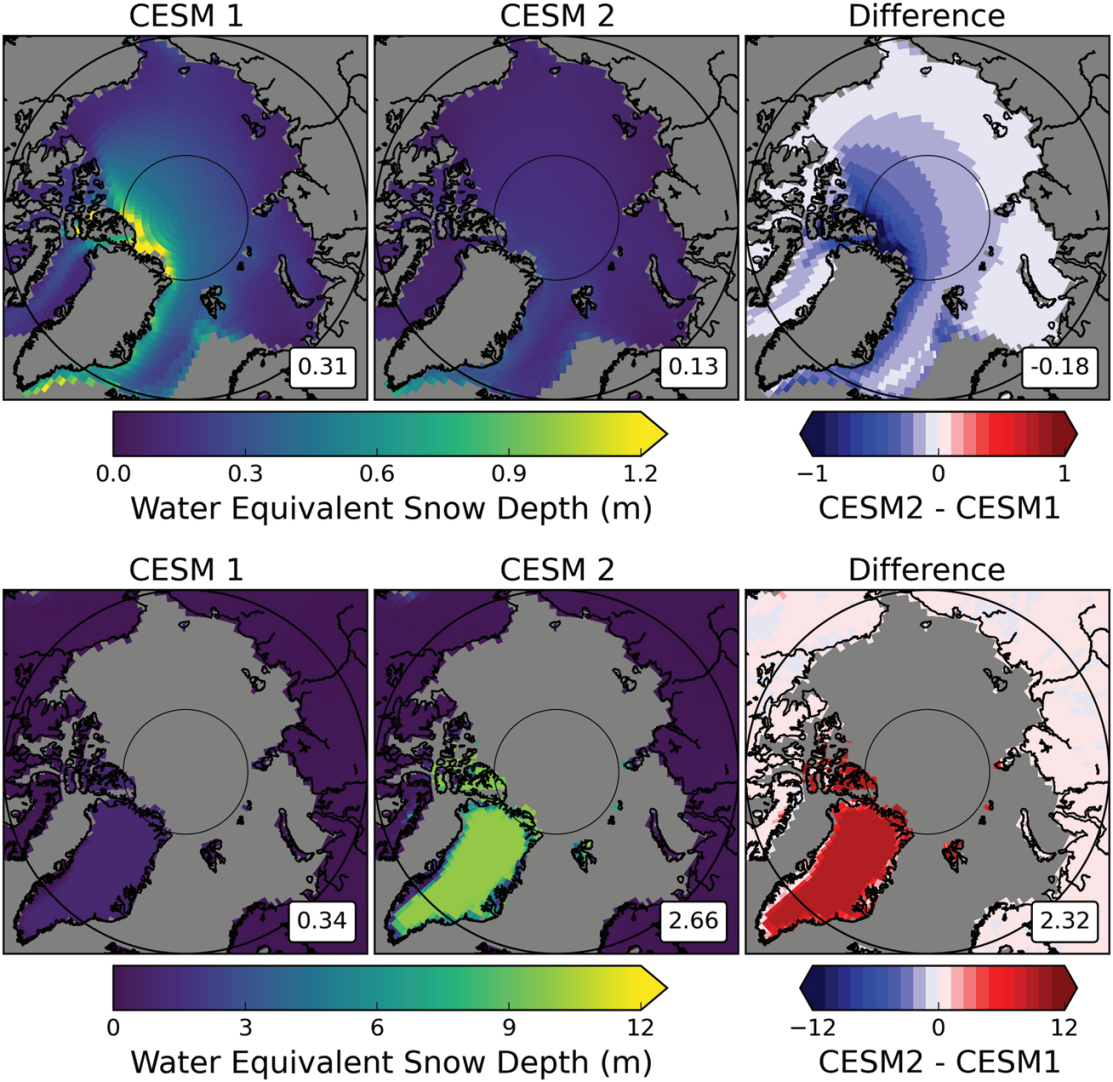
Annual averages of water equivalent snow depth on sea ice (top row) and land (bottom row). The plots for CESM1 (left column) and CESM2 (center column) are means from their respective 800‐year control runs. The difference plots in the right column are CESM2 minus CESM1, with red (blue) values showing increases (decreases) in snow depth in CESM2 with respect to CESM1. The area‐weighted averages for the study area (66.91°N and 90°N) are shown in the lower right of each map.

Given the slight increase in Arctic annual precipitation total going from CESM1 to CESM2, we would expect a coinciding increase for the snowpack on Arctic sea ice (monthly mean “SNOWHICE” where “ICEFRAC” ≥0.5). However, we find that the snowpack on sea ice in CESM2 is consistently shallower than CESM1 (Figure 9, top row). CESM1 has an annual mean water equivalent snow depth of 0.31 m, whereas CESM2 has 0.13 m. The seasonal plots of snow pack are qualitatively similar to the annual mean, thus are not included here. The largest differences in snow on sea ice are centered around the north and northeastern coastlines of Greenland, stretching toward the north pole (Figure 9, top right). This region is also where the snow depth is deepest in CESM1 (Figure 9, top left), with a maximum grid box mean value of 4.31 m. In CESM2, the snow depth over sea ice is relatively low and uniform, with no grid box mean values above 0.40 m.

While the mean snowpack on sea ice has undoubtedly decreased in CESM2, the snowpack on Arctic land surfaces (“SNOWHLAND” where “LANDFRAC” ≥0.5, Figure 9, bottom row) has increased in the updated version. The annual mean water equivalent snow depth on land is 0.34 m in CESM1 and 2.66 m in CESM2. The majority of the increase is located over the GrIS (Figure 9, bottom right) where the mean differences include increases in liquid equivalent depth as large as 9.92 m. The temperature and elevation differences between the GrIS and the sea ice could perhaps explain some of the large difference in how the precipitation is interacting with the two icy surfaces. However, in CESM2, nearly all land surfaces in the Arctic have increases in snow depth relative to CESM1, whereas all sea ice surfaces have decreases. There is clearly a difference in accumulation and/or melt behavior between the two surface types in the two models.

#### 3.3.2 Snowfall

**Figure 10:**
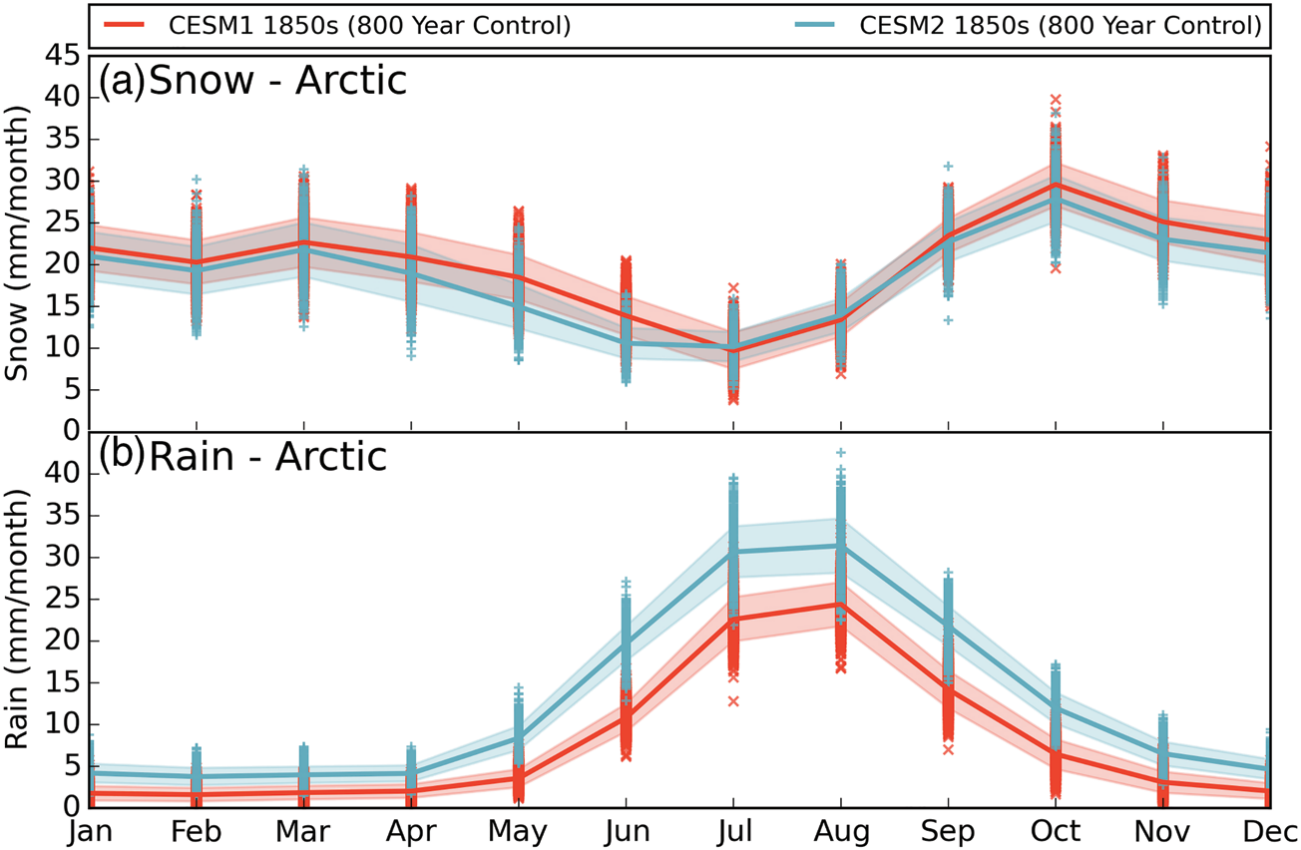
As in Figure 1 for the annual cycles of (a) snowfall rate and (b) rainfall rate. Note the*y*‐axes are the same. Model data are from years 400–1199 of the preindustrial control runs.

To explore changes in the snowpack between CESM1 and CESM2, we must further separate Arctic precipitation into its two components: snow (the sum of monthly mean convective and large‐scale snowfall “PRECSC” + “PRECSL”) and rain (total convective and large scale precipitation [“PRECC” + “PRECL”] minus snowfall [“PRECSC” + “PRECSL”]). The annual cycle of snowfall rates is shown in Figure 10a. Mean Arctic snow rates in CESM2 have decreased in April, May, and June, while remaining similar to CESM1 in all other months. In the annual mean, CESM1 snows 20 mm month^−1^, and CESM2 snows 19 mm month^−1^. The magnitude of the difference is small, but it illustrates a consistent divergence in the mean state of the two models. Over long periods of time and over the full Arctic, a small change in snowfall behavior can lead to large changes in accumulation.

**Figure 11:**
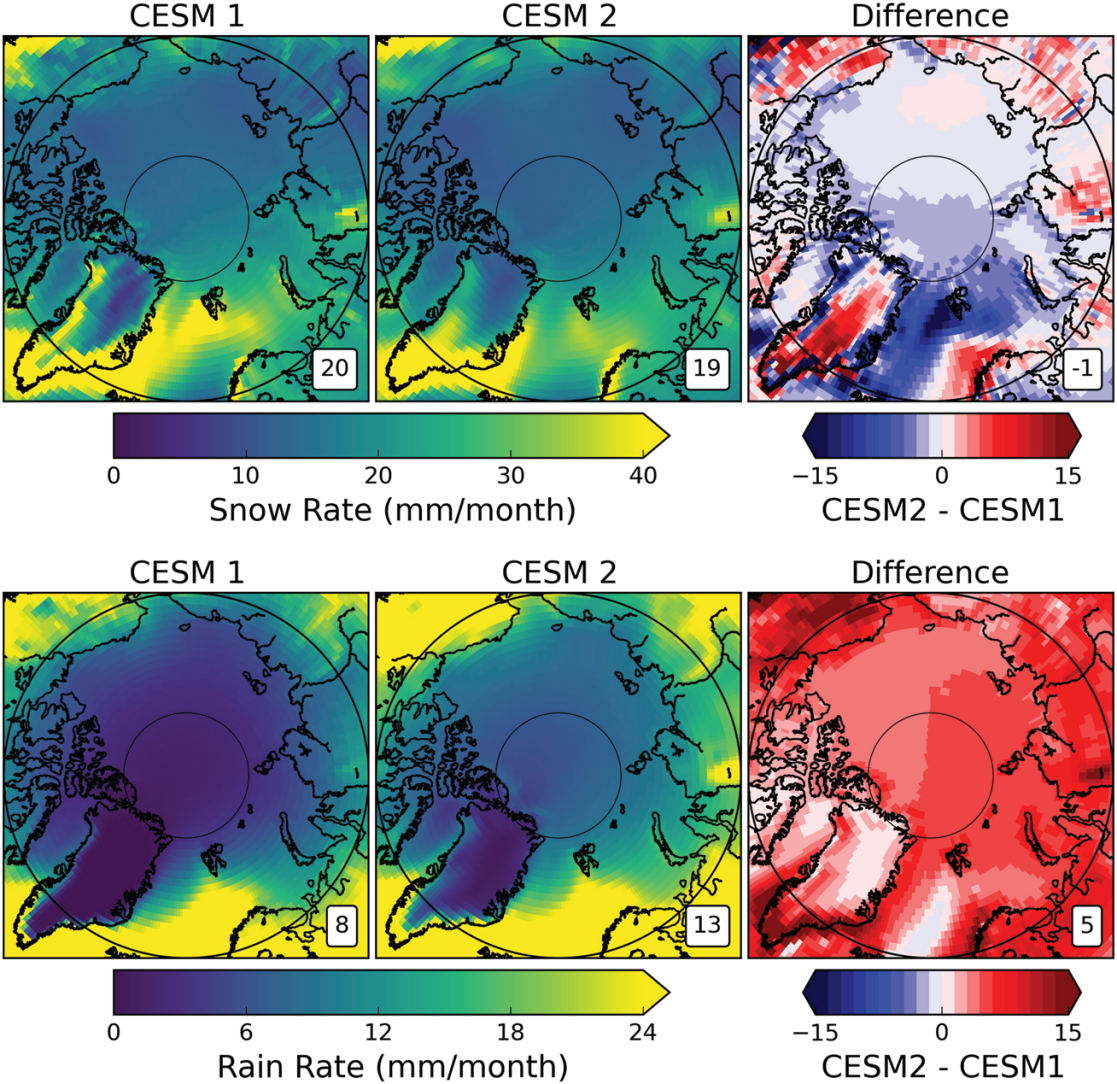
As in Figure 9 for snow rate (top row) and rain rate (bottom row). The plots for CESM1 (left column) and CESM2 (center column) are means from their respective 800‐year control runs.

Looking at the spatial distributions of Arctic snowfall (Figure 11, top row), we see that the differences in snowfall (CESM2‐CESM1) are predominantly negative over the open ocean and sea ice, while they are generally positive over land with most of the large positive changes occurring over the central GrIS. This opposite sign change based on surface type fits with the differing snowpack changes discussed in section 3.3.1. There are regional differences in the snowpack changes that cannot be explained by snowfall rates alone. For example, the snow rate changes over the GrIS and Alaska are similar; however, the increase in snowpack over the GrIS is far greater, which could be due to more temperatures near and above freezing in CESM2's Alaska (Figure 7). Knowing that Arctic snowfall has decreased while the total annual precipitation increased in CESM2 relative to CESM1, the difference must be made up for by an increase in Arctic rainfall.

#### 3.3.3 Rainfall

Indeed, mean annual Arctic rainfall has increased from 8 mm month^−1^ in CESM1 to 13 mm month^−1^ in CESM2. Figure 10b shows that CESM2 rainfall has increased in all months, with the largest magnitude changes May–October. Spatially, the rainfall is increased in all of the Arctic, with the largest increases over the southeastern GrIS, northern Alaska, and the Barents Sea (Figure 11, bottom right).

The Arctic surface temperature in CESM2 is higher than CESM1 (discussed in section 3.2.2). However, the winter mean temperatures for both models are still well below freezing (∼247 K), and temperatures over the sea ice are lower still (Figure 7). So why is it raining in CESM2 across the Arctic in winter?

### 3.4 Cloud Liquid Tendencies

**Figure 12:**
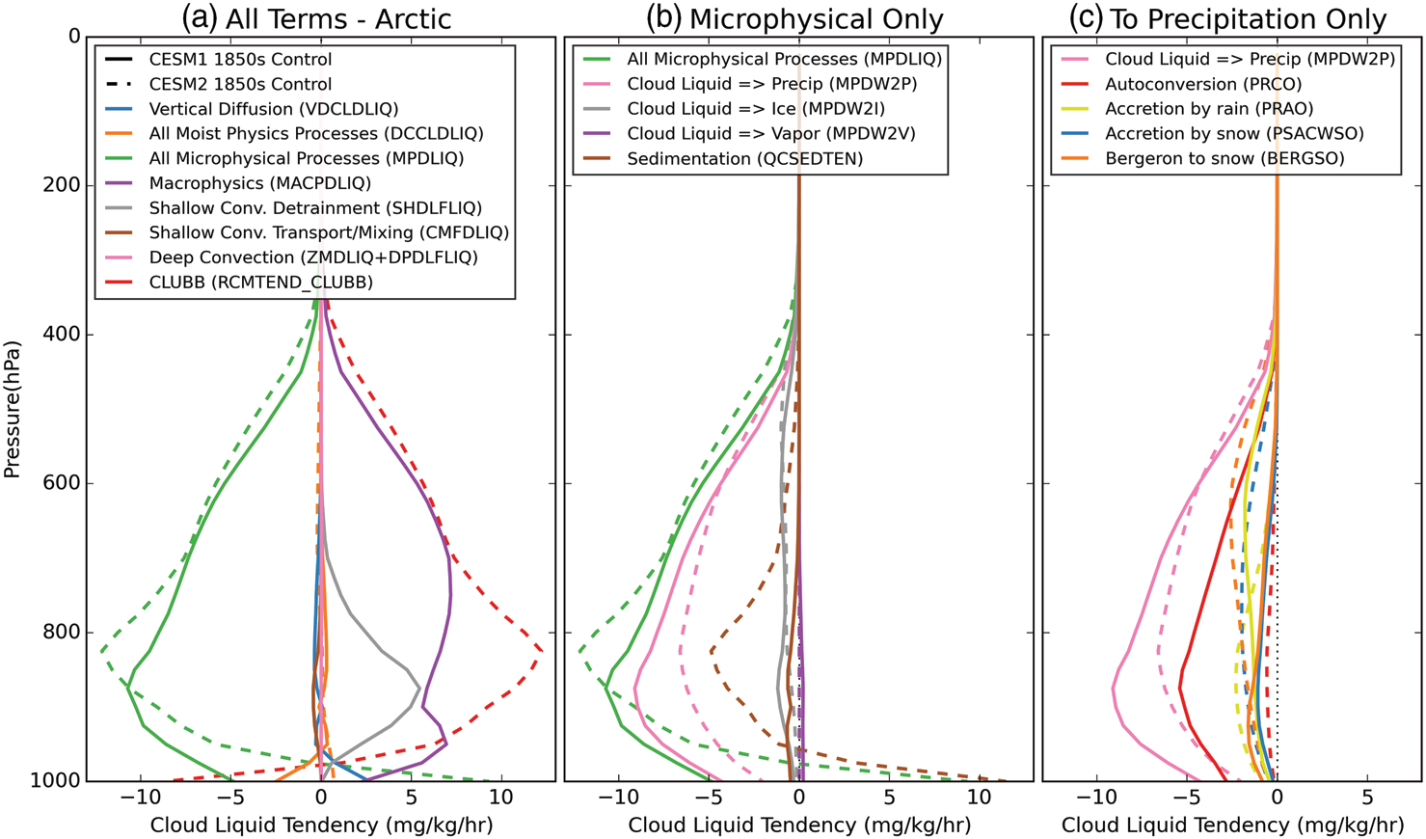
Vertical profiles of annual mean cloud liquid tendencies in the Arctic region (66.91–90°N) for CESM1 (solid lines) and CESM2 (dashed lines) from the 10‐year branch simulations of their respective 1850s control runs. The values are averages of the 10 years of monthly averaged output. (a) contains all moist physics processes (“DCCLDLIQ”) and its component parts CESM1: “DCCLDLIQ” = “MPDLIQ” + “MACPDLIQ” + “SHDLFLIQ” + “CMFDLIQ” + “ZMDLIQ” + “DPDLFLIQ”; CESM2: “MPDLIQ” + “RCMTEND_CLUBB” + “ZMDLIQ” + “DPDLFLIQ”), as well as vertical diffusion (“VDCLDLIQ”). (b) contains the microphysical tendency (“MPDLIQ”) and its component parts (“MPDLIQ” = “MPDW2P” + “MPDW2I” + “MPDW2V” + “QCSEDTEN”). (c) contains the microphysical conversion of cloud liquid to precipitation (“MPDW2P”) and its component parts (“MPDW2P” = −’PRAO” − “PRCO” − “PSACWSO” − “BERGSO”).

Tendency terms show the conversions that create and deplete cloud liquid and have been used previously to explore LCC prevalence in CESM polar regions (Kay, Wall et al., 2016; McIlhattan et al., 2017). Figure 12 shows the mean Arctic vertical profiles for the tendency terms that create and deplete cloud liquid in CESM1 (solid lines), and those same values for CESM2 (dashed lines). The vertical axis is the height in pressure units, and the horizontal is the rate of creation/depletion. The overall tendency of cloud liquid (“DCCLDLIQ”) and variables that contribute to it are included in Figure 12a. In both CESM1 and CESM2, the total tendency of cloud liquid (“DCCLDLIQ”) remains close to zero throughout the column due to the competing tendencies of microphysics (“MPDLIQ”) and large‐scale/dynamic processes (CESM1: “MACPDLIQ,” “SHDLFLIQ,” “‘CMFDLIQ,” “ZMDLIQ,” and “DPDLFLIQ”; CESM2: “RCMTEND_CLUBB,” “ZMDLIQ,” and “DPDLFLIQ”). We have included all of the terms in for completeness (Figure 12a); however, our interest is in precipitation, so we focus on the changes in microphysical processes (“MPDLIQ,” green lines), which is broken down into its components in Figure 12b.

In both CESM1 and CESM2, microphysical processes (“MPDLIQ,” green lines, Figures 12a and 12b) primarily act to deplete cloud liquid from the Arctic. In both model versions, the microphysical removal of cloud liquid is primarily accomplished though conversion to precipitation (“MPDW2P,” pink lines, Figure 12b). The conversion to precipitation has increased slightly above 600 hPa but decreased markedly below that level in CESM2 relative to CESM1, which helps to explain why the large increase in Arctic cloud liquid (section 3.1) is not accompanied by a large increase in precipitation (section 3.3).

While the microphysical processes overall act to remove cloud liquid from the Arctic in both models, there is a stark difference in behavior near the surface. CESM1 microphysics removes liquid in the whole column, but CESM2 microphysical processes actually produce cloud liquid below ∼950 hPa. Sedimentation (“QCSEDTEN,” brown lines, Figure 12b) is entirely responsible for producing the near surface cloud liquid in CESM2. Both model versions consider the sedimentation of cloud liquid, but in CESM1, there is too little cloud liquid for sedimentation to play a large role in the cloud liquid tendency (solid brown line in Figure 12b). Whereas in CESM2, sedimentation acts to remove cloud liquid from the upper levels of the atmosphere, bringing it toward the surface. At the surface, sedimentation in CESM2 produces cloud liquid at a mean rate of ∼11 mg kg^−1^ hr^−1^. Cloud liquid that sediments out of the bottom of the CAM6 atmosphere is converted to a precipitation flux. There is not a specific freezing mechanism for sedimenting condensate; thus, supercooled cloud liquid sedimentation from the bottom model layer is converted to a surface flux of liquid precipitation even in subfreezing temperatures (A. Gettelman, personal communication).

The direct conversion of cloud liquid to precipitation (“MPDW2P”) is a combination of autoconversion (“PRCO,” red lines), accretion by rain (“PRAO,” yellow lines), accretion by snow (“PSACWSO,” blue lines), and conversion to snow by the Bergeron process (“BERGSO,” orange lines). All components have changed somewhat between the two versions, but the dominant change is in autoconversion. In CESM1, autoconversion acted as the primary conversion of cloud liquid to precipitation, but in CESM2, it is the least active of the four processes. This reduction is consistent with the change in autoconversion scheme as described in Gettelman et al. (2019) supplemental information.

The key results from the tendency terms presented in Figure 12 are as follows: first, the direct conversion of Arctic cloud liquid to precipitation is reduced in CESM2 relative to CESM1, and thus the mean snowfall rate is also reduced despite the dramatic increase in cloud liquid; second, due to the increased sedimentation of Arctic cloud liquid in CESM2, the mean Arctic rainfall rate has also increased year‐round, despite subfreezing temperatures.

## 4 Discussion

Our results demonstrate that the Arctic mean states in CESM1 and CESM2 have distinctly different cloud, radiation, and precipitation characteristics. Specifically, we leveraged the 1850s control simulations of each model version to show that the Arctic in CESM2 is cloudier, warmer, and rainier than in CESM1.

Previous studies documented that CESM1 produces too few LCCs relative to observations (Cesana et al., 2015; Kay, Bourdages et al., 2016; Tan & Storelvmo, 2016). While modern era simulations using CESM1 do produce a spatial distribution of Arctic LCCs comparable to observations, their overall LCC frequency is much too low (McIlhattan et al., 2017). In this work, we show that CESM2 has addressed this issue, simulating Arctic LCCs at more than twice the frequency of CESM1. Total cloud water path in the Arctic is more than five times greater in CESM2 than in CESM1. These findings are consistent with the results from Lenaerts et al. (2020), which showed an increase in liquid water path over the GrIS in CESM2 relative to CESM1 and credited that improvement to two updated components: a mixed‐phase ice nucleation scheme and a new version of the microphysics package including prognostic precipitation.

The increase in cloud liquid in CESM2 brings with it the qualitatively expected changes in downwelling surface radiation and temperature: first, an increase in downwelling LW radiation (+22 W m^−2^) due to enhanced trapping of outgoing terrestrial radiation; and second, a decrease in downwelling SW radiation (−23 W m^−2^) due to increased reflection of incoming solar. Between the competing effects, the SW is slightly stronger, which leads to a slight decrease in total downwelling radiation in the newer model version. However, the land and sea ice surfaces of CESM2 are less reflective and the radiation actually absorbed at the surface is 16 W m^−2^ greater in CESM2 compared to CESM1. This is consistent with decreased sea ice cover, decreased snow cover, and/or changes in land, ice, and snow albedo. Karlsson and Svensson (2013) showed a connection between model parameterized sea ice albedo and the magnitude of summer sea ice melt in CMIP5 models, indicating that reduced sea ice albedo can lead to larger summer melt and thus additional SW radiation absorption. The increase in absorbed radiation in CESM2's Arctic is likely largely responsible for the increased mean Arctic surface temperatures (+3 K) relative to CESM1.

While it is important and needed progress for CESM2 to produce more Arctic LCCs, our analysis shows some evidence that CESM2 might now be overproducing cloud liquid relative to what is reasonable, particularly in summer. When compared with the mean LCC frequency from present‐day observations (0.45), we find that the historical control run of CESM2 produces LCCs more frequently in the annual average (0.52). The increased frequency relative to observations occurs predominantly in the summer months (June–August), a time when the total amount of cloud liquid is consistent in CESM2 between preindustrial and modern eras. In these same months, we see a large reduction in CESM2's downwelling SW radiation, which shifts the peak values to May. Both CESM1 and the observations have the peak in downwelling SW radiation in June, coincident with peak solar insulation at the top of atmosphere. Despite the differing time periods, both CESM versions should likely have the same shape of radiation distribution, even if the magnitude is lower. The mismatch in SW is seen in the total downwelling radiation as well. In comparing modern era simulations with satellite observations, Lenaerts et al. (2020) found that CESM2 slightly overestimates liquid water path over the GrIS in summer months. Our study has a mismatch of time periods, and the Lenaerts et al. (2020) study uses a single model realization to compare to observations, neither setup is able to say whether or not the observations fit within the intramodel variability for the observational years. A large ensemble is planned for CESM2, which will provide an opportunity to see if the observed values fit within the model's internal variability or if indeed Arctic clouds have too much liquid in the updated version.

McIlhattan et al. (2017) suggested that the low bias in Arctic LCC frequency in CESM1's modern era simulations could be due to an overactive conversion of cloud liquid to snowfall. With the drastic increase in cloud liquid we found in CESM2, we expected that perhaps the precipitation frequency in LCCs would be reduced relative to CESM1. We instead found that CESM2's modeled LCCs were still precipitating at nearly the same frequency as in CESM1 (∼0.65), much more frequently than is reasonable, based on observations (∼0.13). Total precipitation rate and frequency are increased in CESM2 compared to CESM1 (+3 mm month^−1^, +0.08, respectively). Overall, CESM2 is both precipitating more often and with lighter events than CESM1. Many GCMs, including CESM1, have a notable history of precipitating too lightly, too often (Dai, 2006; Kay et al., 2018; Stephens et al., 2010; Terai et al., 2018), so it comes as a surprise that the updated version of CESM has more frequent precipitation, rather than less.

Less snow falls in the CESM2 Arctic than the CESM1 Arctic, with tendency terms showing the direct conversion of cloud liquid to snowfall has decreased. This should have at least partially addressed the issue of overactive snowfall in CESM1 LCCs identified by McIlhattan et al. (2017). However, an increase in cloud liquid sedimentation in CESM2 has resulted in more rain, even in subfreezing winter temperatures, maintaining the too high precipitation frequency in LCCs. There is observational evidence that supercooled drizzle does occur in the Arctic (Cortinas Jr. et al., 2004; Kajikawa et al., 2000; Wood, 2012); however, the observations are limited in time and space and do not provide a broad climatology to determine if the below freezing rainfall in the CAM6 Arctic is occurring at a reasonable rate or frequency. Locally, however, Lenaerts et al. (2020) found that CAM6 simulated excessive rainfall over the coastal GrIS in the modern era relative to observationally validated regional model data, which is likely linked to the CAM6 cloud liquid sedimentation issue we present here.

DuVivier et al. (2020) found that CESM2‐CAM6 modern era simulations substantially underestimate both Arctic sea ice volume and sea ice extent relative to observationally based estimates. Similarly, DeRepentigny et al. (2020) found thinner ice in CESM2‐CAM6 than both observations and CESM1 estimates. The reduction in CESM2 sea ice is consistent with our findings that the Arctic mean state in CESM2 has warmer surfaces, reduced snowfall, reduced snowpack, and increased rain over the sea ice region, compared to CESM1.

Conversely, we have found that the land surfaces of the CESM2 Arctic have increased snowfall and snowpack relative to CESM1. Lenaerts et al. (2020) found that in simulations of the modern era, central GrIS surface melt is similar but slightly stronger in CESM1 than CESM2, which, combined with the increase in snowfall, could explain the dramatic increase in snowpack (approximately +10 m liquid water equivalent).

In this work, we chose to compare 1850s preindustrial control simulations of the two model versions in order to focus on changes in the mean state of modeled cloud and precipitation. Gettelman et al. (2019) showed that cloud feedbacks in CESM2 have led to an increased equilibrium climate sensitivity, meaning that a doubling of CO_2_ has a larger impact on surface temperatures in CESM2 than CESM1. With both the increased mean Arctic temperature in CESM2 (+3 K) and the increased climate sensitivity, it is reasonable to extrapolate that the CESM2 Arctic will be more sensitive to greenhouse gas emissions and perhaps see greater magnitude warming in future projections. The upcoming large ensemble will allow this hypothesis to be explored in greater detail.

The Earth science community has known that the Arctic is a focal point for global warming for more than a century. As GCMs improve, including more detailed physical processes and realistic parameters, modern‐day representations approach observational values and our confidence in future projections rises (Knutti et al., 2013). CESM2 simulates a different Arctic than CESM1; it has addressed the long‐standing issue of too few LCCs, but also produces wintertime rain in locations that are not physically reasonable. In a model as complex and interconnected as CESM, progress is not likely to be simple or linear, but the trajectory is toward a better understanding of the Earth's climate.

## 5 Summary and Conclusions

We documented changes in Arctic clouds and precipitation in the newly released CESM2‐CAM6, relative to the previous version (CESM1‐CAM5). We used 1850s preindustrial control and branch simulations in order to focus on changes in the mean state, as opposed to changes resulting from differing transient forcings. Compared to CESM1, the CESM2 Arctic exhibits the following:

- Increased LCC frequency and total cloud liquid,
- Increased surface downwelling LW radiation,
- Decreased surface downwelling SW radiation,
- Decreased surface albedo,
- Overall reduced downwelling radiation at the surface, though total absorbed radiation has increased,
- Increased surface temperature,
- More frequent precipitation,
- Less snow over the sea ice and more snow over land,
- More rain everywhere, and
- Decreased snowpack on sea ice while snowpack on land has increased, particularly on the GrIS.

Broadly, this work demonstrates that the CESM2 Arctic has undergone major changes relative to CESM1. The mean state in CESM2 is cloudier, warmer, and rainier. Future work will utilize the upcoming CESM2 large ensemble to further investigate Arctic cloud and precipitation representations and their influence on transient climate change and variability.

## Supporting information



Supporting Information S1

## Data Availability

The CESM data used in this study can be downloaded from NCAR Campaign Storage using Globus using the following links: 10‐year branch simulations (CESM1: /glade/campaign/cesm/development/pcwg/jenkay/ cesmle_B1850_COSP1p4_PCWG3; CESM2: /glade/campaign/cesm/development/pcwg/jenkay/ cesm2p1_B1850_COSP2_PCWG2), preindustrial control runs (CESM1: /glade/collections/cdg/data/cesmLE/CESM‐CAM5‐BGC‐LE/; CESM2: /glade/campaign/collections/cmip/CMIP6/timeseries‐cmip6/b.e21.B1850.f09_g17.CMIP6‐piControl.001/), and historical ensemble members (CESM1: /glade/collections/cdg/data/cesmLE/CESM‐CAM5‐BGC‐LE/; CESM2: /glade/campaign/collections/cmip/CMIP6/timeseries‐cmip6/ b.e21.BHIST.f09_g17.CMIP6‐historical.0**/). For more information on using Globus on NCAR systems, please refer online (https://www2.cisl.ucar.edu/resources/storage‐and‐file‐systems/globus‐file‐transfers). The satellite derived data used in this analyses are publicly available from the CloudSat Data Processing Center (http://www.cloudsat.cira.colostate.edu/data‐products).
