# Supplementary material for: Arctic Clouds and Precipitation in the Community Earth System Model Version 2
Source: J Geophys Res Atmos. 2020 Nov 20;125(22):e2020JD032521. doi: 10.1029/2020JD032521 (PMC7757258; doi:10.1029/2020JD032521)
Supplement: Supplementary file 1 — Supporting Information S1 [file JGRD-125-e2020JD032521-s001.pdf]

Supporting Information for "Arctic Clouds and Precipitation in the Community Earth System Model Version 2"

Elin A. McIlhattan[1], Jennifer E. Kay[2], Tristan S. L'Ecuyer[1]

[1]Department of Atmospheric and Oceanic Sciences, University of Wisconsin-Madison, Madison, Wisconsin, USA

[2]Department of Atmospheric and Oceanic Sciences, University of Colorado, Boulder, CO, USA

Contents of this file

1. Comparing CESM Branch and Control Simulations
2. Figure S1
3. Table S1

Comparing CESM Branch and Control Simulations

To demonstrate the representative nature of the branch simulations, we include both the control runs (800 years, gray lines) and the branch simulations (10 years, colored lines) for total cloud water in Fig S1, as well as their annual means in Table S1. While the variability shown by the individual years of the control runs is larger than for the branch simulations, the branch simulations capture the annual mean as well as the annual cycle of the control runs for Arctic total cloud water. Comparisons between the control and branch simulations for all other standard variables (not shown) have similar results,

giving us confidence that the branch simulations are largely representative of the longer term Arctic mean state.

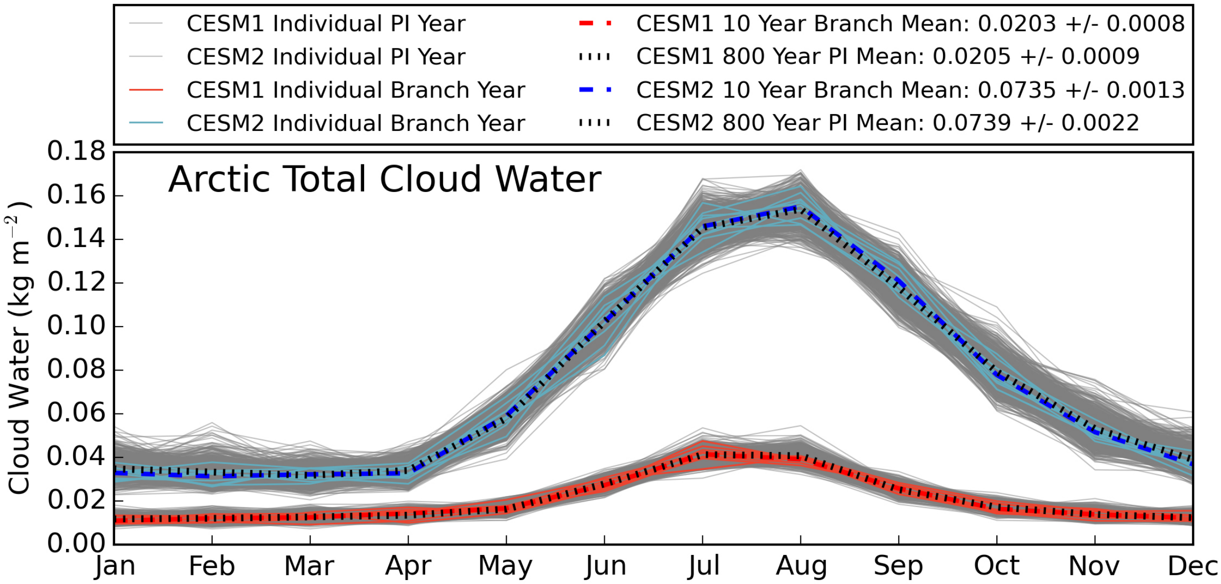

Figure S1. A comparison of the 800 year pre-industrial control run data and associated 10 year branch simulations for Arctic total cloud water (monthly mean 'TGCLDLWP'+'TGCLDIWP'). Individual years for both pre-industrial control runs are plotted in grey. Individual branch years are plotted in red for CESM1 and blue for CESM2. The monthly mean for the control runs are shown with black dotted lines while the branch runs are shown with dashed colored lines. The variability is larger for the controls than the branches, however the long term means are well captured by the branch runs.

Table S1. Comparison of annual mean values for total cloud water. The ± value is the standard deviation of the annual mean values. The "1850s Control" is comprised of 800 years of the pre-industrial control simulation. The "1850s Branch" is the 10 year branch simulation run from the pre-industrial control. The values are area weighted means for the full modeled Arctic (66.91-90° N).

**Dataset**	**Spatial Region**	**Time Period**	**Total Cloud Water** (kg m^{-2})
CESM1	67-90° N	1850s Branch	0.0203 ± 0.0008
CESM1	67-90° N	1850s Control	0.0205 ± 0.0009
CESM2	67-90° N	1850s Branch	0.0735 ± 0.0013
CESM2	67-90° N	1850s Control	0.0739 ± 0.0022
